# Human cardiac fibroblasts expressing VCAM1 improve heart function in postinfarct heart failure rat models by stimulating lymphangiogenesis

**DOI:** 10.1371/journal.pone.0237810

**Published:** 2020-09-16

**Authors:** Takahiro Iwamiya, Bertrand-David Segard, Yuimi Matsuoka, Tomomi Imamura

**Affiliations:** 1 Research & Development Department, Metcela Inc., Kawasaki, Kanagawa, Japan; 2 Institute for Advanced Biosciences, Keio University, Tsuruoka, Yamagata, Japan; Scuola Superiore Sant'Anna, ITALY

## Abstract

Cardiovascular diseases are a leading cause of death worldwide. After an ischemic injury, the myocardium undergoes severe necrosis and apoptosis, leading to a dramatic degradation of function. Numerous studies have reported that cardiac fibroblasts (CFs) play a critical role in heart function even after injury. However, CFs present heterogeneous characteristics according to their development stage (i.e., fetal or adult), and the molecular mechanisms by which they maintain heart function are not fully understood. The aim of this study is to explore the hypothesis that a specific population of CFs can repair the injured myocardium in heart failure following ischemic infarction, and lead to a significant recovery of cardiac function. Flow cytometry analysis of CFs defined two subpopulations according to their relative expression of vascular cell adhesion molecule 1 (VCAM1). Whole-transcriptome analysis described distinct profiles for these groups, with a correlation between VCAM1 expression and lymphangiogenesis-related genes up-regulation. Vascular formation assays showed a significant stimulation of lymphatic cells network complexity by VCFs. Injection of human VCAM1-expressing CFs (VCFs) in postinfarct heart failure rat models (ligation of the left anterior descending artery) led to a significant restoration of the left ventricle contraction. Over the course of the experiment, left ventricular ejection fraction and fractional shortening increased by 16.65% ± 5.64% and 10.43% ± 6.02%, respectively, in VCF-treated rats. Histological examinations revealed that VCFs efficiently mobilized the lymphatic endothelial cells into the infarcted area. In conclusion, human CFs present heterogeneous expression of VCAM1 and lymphangiogenesis-promoting factors. VCFs restore the mechanical properties of ventricular walls by mobilizing lymphatic endothelial cells into the infarct when injected into a rat heart failure model. These results suggest a role of this specific population of CFs in the homeostasis of the lymphatic system in cardiac regeneration, providing new information for the study and therapy of cardiac diseases.

## Introduction

Coronary artery diseases are a leading cause of morbidity and mortality worldwide. Following ischemia, affected myocardial tissues undergo massive remodeling including apoptosis of resident cells and replacement by non-contractile structures in an attempt to prevent wall rupture. The electromechanical properties of the resultant scar lead to degraded functionality of the whole organ, and eventually to heart failure. The restoration of cardiac function thus requires alteration of both the tissue structure and its electromechanical properties.

Various strategies have been developed to respond to this clinical challenge, including the promotion of angiogenesis [1], transplantation of progenitor cells [2], transplantation of embryonic stem (ES)/induced pluripotent stem (iPS) cell-derived cardiomyocytes [3], induction of transdifferentiation of resident cells into cardiomyocytes [4], and reprogramming of stem cells [5]. Despite impressive progress, these methods have generated limited clinical impact. Their low clinical efficacy might be explained by the focus of these strategies on mobilizing cardiomyocytes while neglecting complementary aspects such as restoring the electromechanical properties of the ventricular wall or the local structure of the circulatory system. This situation calls for an innovative therapeutic approach to restore myocardial function in patients with heart failure.

Among resident cells of the myocardium [e.g., cardiac fibroblasts (CFs), cardiomyocytes, endothelial cells, and vascular smooth muscle cells], CFs are particularly critical for maintaining heart function in health and disease [6,7]. They participate in the structural organization of the tissue by regulating extracellular matrix (ECM) homeostasis and modulating the proliferation/cell death balance, autophagy, adhesion, and migration of numerous other types of cells [8]. The myocardial ECM plays a central role in the distribution of signaling and structural proteins, and extensively influences the behavior of all cardiac cells [9]. Thus, CFs significantly influence electrophysiological properties, secretion of growth factors and cytokines, and blood vessel formation in the myocardium [10]. Based on this evidence, the capacity of CFs to interact with other cells and modulate the overall tissue characteristics has been successfully used in myocardium bioengineering [11,12]. Moreover, in cardiac tissue engineered from induced pluripotent stem cells, the presence of CFs is mandatory for the establishment of functional grafts for cardiac regenerative therapies [13].

By contrast, in pathological states, keloid-like translocation of cardiac fibroblasts is the leading cause of cardiac fibrosis in the infarcted necrotic tissue. Progressive increase in this process can disrupt systolic heart function and lead to left ventricular hypertrophy. Moreover, CFs respond to numerous factors, including proinflammatory cytokines (i.e., TNF-α, IL-1, IL-6, TGF-β), platelet-derived growth factor (PDGF), connective tissue growth factor, vasoactive peptide systems [particularly angiotensin II and endothelin-1 (EDN-1), as well as natriuretic peptides], and hormones (e.g., noradrenaline). CFs also promote cardiac remodeling and heart failure via blood pressure elevation, cardiomyocyte apoptosis, and inflammation [6,14,15]. These processes involve interactions with T lymphocytes as regulators of the cardiofibroblast/myofibroblast transition and subsequent ECM remodeling [16].

In this manner, CFs make “positive” and “negative” contributions to cardiac development, repair, and pathogenesis. However, the CF-derived factors that maintain myocardial function have not yet been described precisely. Moreover, since CFs originate from several sources (e.g., myocardial niche, circulation, and epithelial-to-mesenchymal transition) [10,17], they constitute a heterogeneous population and therefore have a wide variety of functions. Identifying these CF subpopulations and characterizing their roles in cardiac physiology is essential not only for understanding heart development and pathogenesis but also for developing therapies.

The present study aimed to demonstrate the critical role of a specific population of CFs expressing vascular cell adhesion molecule 1 (VCAM1; VCFs) in the restoration of cardiac function in heart failure following myocardial infarction. It is well known that angiogenesis restores cardiac function after myocardial infarction [1]. Additionally, numerous studies have reported that cardiac lymphatic vessels play a key role in maintaining fluid homeostasis, which is critical for healthy heart contraction [18]. Recently, targeting cardiac lymphatic vasculature is thought to provide a therapeutic benefit to heart function after myocardial infarction [19,20]. Thus, we hypothesized that VCFs might have the potential to improve cardiac function in heart failure by triggering angiogenesis and/or lymphangiogenesis. Therefore, CFs were distinguished and characterized based on their relative expression of VCAM1. VCFs were injected into a postinfarct heart failure rat model, and their influence on cardiac tissue structure and organization over 18 weeks was successfully identified.

## Materials and methods

The authors declare that all supporting data are available within the article and Online Data Supplement. Raw data are available on FigShare at https://figshare.com/projects/Supporting_data_for_PLOS_ONE_Iwamiya_2020_/80726.

### Antibodies and reagents for magnetic-activated cell sorting (MACS), fluorescence-activated cell sorting (FACS), vascular formation assay, and immunohistochemistry

The antibodies and reagents used in MACS and FACS, staining, vascular formation assay, and immunohistochemistry are listed in **S1 Table**.

### Expansion of human fetal CFs and isolation of VCFs and VCAM1-negative CFs (VNCFs)

Human fetal CFs were purchased from Cell Applications (San Diego, CA) and cultured with HFDM-1(+) medium (Cell Science & Technology, Osaka, Japan) supplemented with 1% (v/v) Newborn Calf Serum (NBCS). After initial expansion (passage 3–5), fibroblasts were incubated with a biotin-conjugated anti-CD106 (VCAM1) antibody and anti-biotin microbeads for the isolation of VCFs and VNCFs by MACS (autoMACS Pro Separator, Miltenyi Biotec; using the Posseld2 protocol).

### Immunoprofiling, staining, and immunohistochemistry

#### Characterization of cells

Cells were fixed in 4% paraformaldehyde and kept in phosphate-buffered saline (PBS, Fujifilm Wako Pure Chemical) before incubation with primary and secondary antibodies (for 30 min each). Cells were characterized by FACS for various known markers of fibroblasts, cardiomyocytes, mesenchymal stem cells (MSCs), cardiac stem cells (CSCs), epicardium, and endothelium. Regarding FACS analysis of proteins localized in the cytoplasm, cells were permeabilized with 0.1% saponin (Nacalai Tesque, Kyoto, Japan) in PBS for 15 min after fixation and before incubation with primary antibodies. FACS analyses were performed with a MACSQuant Analyzer following the manufacturer’s instructions (Miltenyi Biotec).

#### Characterization of tissues

Heart sections were fixed with formalin and embedded in paraffin. The tissue structure and organization were observed using hematoxylin and eosin (H&E) stain. Collagen fibers were stained with Sirius Red (SR). The extent of fibrosis was measured by the ratio of the area of SR-stained fibers to the area of H&E-stained tissues (whole section), using HALO software following the manufacturer’s instructions (Indica Labs, Albuquerque, NM). The density of blood vessels in fibrous regions was estimated by immunostaining of von Willebrand Factor (vWF). The number of blood vessels stained in fibrous areas was counted with the software NDP.view+ following the manufacturer’s instructions (Hamamatsu Photonics, Shizuoka, Japan).

For immunohistochemical staining, paraffin heart sections were triple stained with cardiac troponin T (cTnT), prospero-related homeobox 1 (Prox-1), and 4’,6-diamidino-2-phenylindole (DAPI). Images were acquired using an IN Cell Analyzer 2200 (GE Healthcare, Princeton, NJ) and an FV1200 confocal microscope (Olympus, Tokyo, Japan). Prox-1-positive cells in the infarcts (i.e., cTnT-negative regions) of each sample were counted using ImageJ.

### Whole transcriptome analysis

The RNA sequencing procedure was outsourced to GENEWIZ (South Plainfield, NJ), and the subsequent analysis was carried out in-house. RNA-seq data are available on ArrayExpress at https://www.ebi.ac.uk/arrayexpress/experiments/E-MTAB-9161. The whole transcriptome analysis procedure is described in **S1 Text**. Principal component analysis (PCA) data are available on GitHub at https://github.com/Metcela-Code/PCA/releases/tag/v1.0.

### Endothelial and lymphatic endothelial vascular formation assay

To perform endothelial tube formation assay, human umbilical vein endothelial cells (HUVECs) were purchased from PromoCell (Heidelberg, Germany) and cultured with the EGM-2 BulletKit (Lonza, Basel, Switzerland). After initial expansion (3–5 passages), we assessed the direct effects of fibroblasts on angiogenesis by co-culturing two types of fibroblasts (i.e., VCF and VNCF) with endothelial cells as previously reported [21]. Briefly, HUVECs were co-cultured with each type of fibroblasts at a ratio of 1:12 using the EGM-2 BulletKit (HUVECs = 2.0 × 10^4^ cells/cm^2^; Fibroblasts = 2. × 10^5^ cells/cm^2^). After 3 days, co-cultures were fixed with 4% paraformaldehyde and immunostained with antibodies listed in **S1 Table**. Images were acquired using an IN Cell Analyzer 2200.

To perform lymph endothelial tube formation assay, human cardiac microvascular endothelial cells (HMVEC-Cs) were purchased from Lonza (Basel, Switzerland) and cultured with the EGM-2MV BulletKit (Lonza). After initial expansion (3–5 passages), HMVEC-Cs were co-cultured with each type of fibroblasts at the same ratio as that in the endothelial tube formation (1:12) using the EGM-2MV BulletKit. After 3 days, co-cultures were fixed with 4% paraformaldehyde and immunostained with antibodies listed in **S1 Table**. Images were acquired using an IN Cell Analyzer 2200.

The topography of tubes in the micrographs was analyzed using the macro “Angiogenesis Analyzer for ImageJ” (v. 1.0.c, Gilles Carpentier, 2012; available online: http://imagej.nih.gov/ij/macros/toolsets/Angiogenesis%20Analyzer.txt). Briefly, in Icy (v. 2.0.3.0; BioImage Analysis Lab, Institut Pasteur), the channel of interest was extracted, and the background was subtracted. Discontinuities of cell membranes due to staining were corrected by applying an anisotropic PDE-based filter (Icy plugin: “Membrane Filter”, v. 0.1.0.1) [22]. Then, the ROI was defined by enhancing and normalizing the contrast of the filtered images and subtracting the background. Finally, the resulting image was analyzed with the Angiogenesis Analyzer macro. The detailed protocol is available online at dx.doi.org/10.17504/protocols.io.bhtaj6ie.

### Animal experiments

Animal experiments were performed by LSI Medience (Tokyo, Japan), and were approved by the Animal Experiment Committee (Approval number: 2018–1002). F344/N Jcl-rnu/rnu rats were purchased from Japan SLC (Shizuoka, Japan). All efforts were made to minimize animal suffering. The experimental procedure is described in **S2 Text**. Briefly, myocardial infarction was induced in male rats (9 weeks old, 133.4–175.0 g) by 30-min occlusion of the left anterior descending artery as previously reported [23,24]. One week after the operation, the cardiac function baseline was acquired by M-mode echocardiography, and rats presenting a left ventricular ejection fraction (LVEF) inferior to 55% were considered representative of heart failure. With an anticipated mean LVEF of 70% [intermediate between the normal value (90%) and heart failure models (LVEF: 50 ± 5%)] and an anticipated mean left ventricular fractional shortening (LVFS) of 37.5% [intermediate between the normal value (55%) and heart failure model (20 ± 5%)], as well as using the type I (α = 0.05) and type II (β = 0.1) error rates, the minimal size of each experimental group was calculated to be two subjects. Here, groups included at least four animals.

The rats underwent a cell injection protocol with a new thoracotomy as previously reported [25]. Animals were distributed into three groups following different experimental protocols: injection of human VCFs (+ VCF; 2 × 10^6^ cells in 50 μl of DMEM supplemented with NBCS at 10% v/v; n = 4), injection of vehicle (control group; 50 μl of DMEM supplemented with NBCS at 10% v/v; n = 4), and a group which had open heart surgery without ischemia/reperfusion injury and also received no injection (sham; n = 6). As infarcts were large, doses were delivered to two positions on the border of the scar. After injection, cardiac function was monitored by echocardiography every two weeks for 18 weeks. At the end of the monitoring period, animals were sacrificed and tissue samples were collected.

### Data analysis

Data are presented as the mean ± standard deviation (SD) or mean ± standard error (SE). The statistical significance of the difference between two groups was determined by Student’s *t*-test. Because the three experimental groups are of different sizes and unknown distributions, statistical analysis was performed using the non-parametric Kruskal–Wallis test, followed by Dunn’s post hoc test. Computation was performed in R (v3.5.1).

## Results

### VCAM1 expression distinguishes two subpopulations of CFs

**Fig 1:**
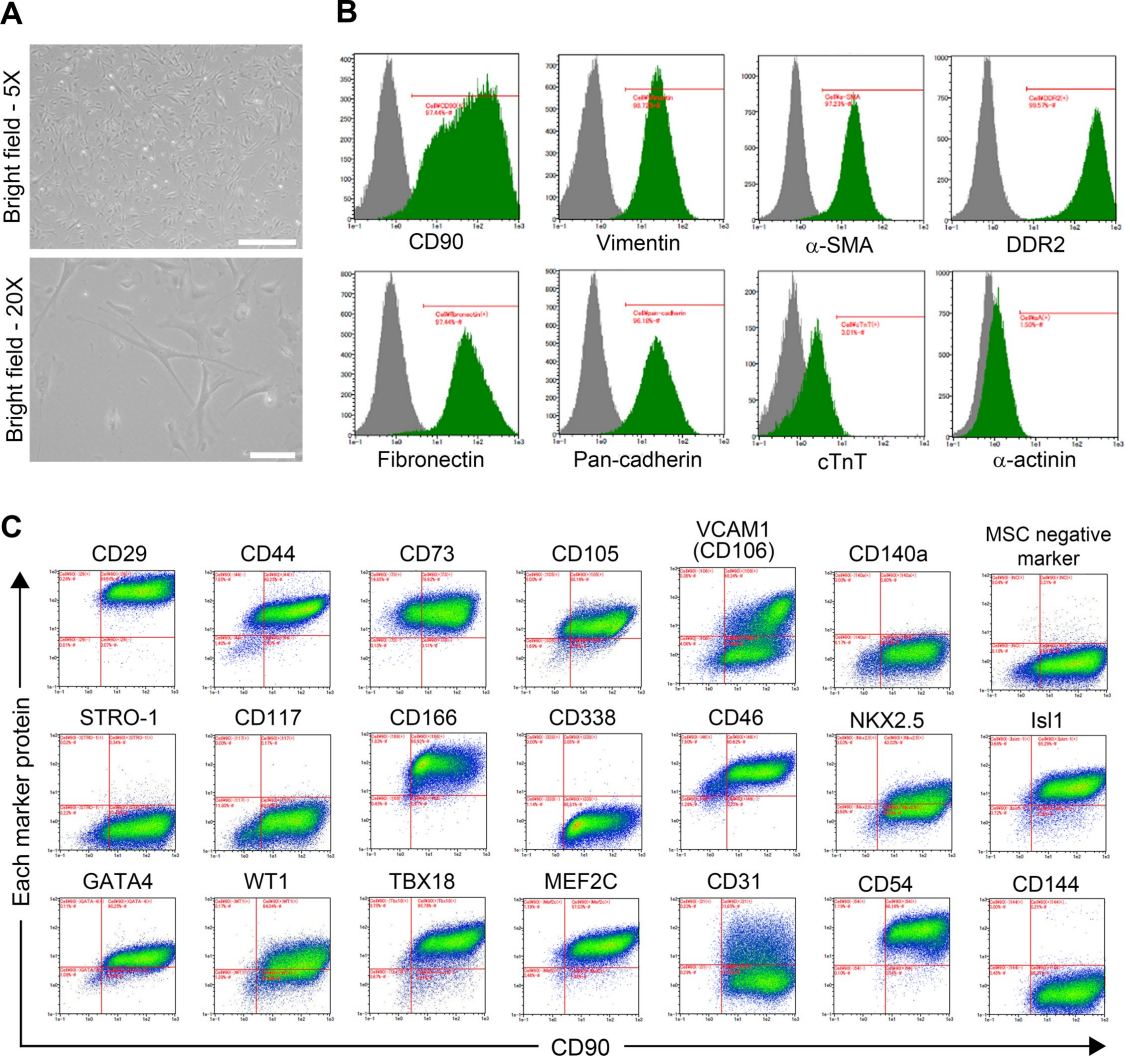
Characterization of CFs. **A,** Micrographs of plated CFs cultured for five days (scale bars: 500 μm (5X) and 100 μm (20X)). **B,** Cell immunoprofiling showing the expression of several markers of fibroblasts (i.e., CD90, vimentin, α-SMA, DDR2, fibronectin, and pan-cadherin) and cardiomyocytes (i.e., cTnT and α-actinin). **C,** Cell immunoprofiling revealing the expression of MSC markers (i.e., CD29, CD44, CD73, CD105, VCAM1, CD140a, STRO-1, CD117, and CD166), CSC (i.e., CD338, NKX2.5, Isl1, and GATA4), epicardium and epicardium-derived cells (EPDC; i.e., CD46, WT1, TBX18, and MEF2C), and endothelial cell markers (i.e., CD54, CD31, and CD144) plotted against CD90 expression.

Human fetal CFs were cultivated onto 10 cm-diameter dishes for initial expansion. CFs exhibited a flat and spindle-shaped morphology typical of the fibroblastic phenotype (**Fig 1A**). CFs strongly expressed fibroblast markers: CD90 in 97.36 ± 0.14% of cells, vimentin in 98.70 ± 0.06%, α-SMA in 97.19 ± 0.15%, DDR2 in 99.62 ± 0.04%, fibronectin in 97.47 ± 0.04%, and pan-cadherin in 96.06 ± 0.15% (n = 3). Conversely, very few cells expressed cardiomyocyte markers cTnT or α-actinin (2.52 ± 1.33% and 1.35 ± 0.40%, respectively; n = 3), ruling out the presence of cardiomyocytes in this population (**Fig 1B)**.

Additional characteristics of CFs were defined by high-throughput cell profiling for the co-expression of CD90 and various MSC markers [26–28]: integrin subunit beta 1 (CD29), CD44, 5’-nucleotidase ecto (CD73), endoglin (CD105), vascular cell adhesion molecule 1 (VCAM1, CD106), PDGF receptor alpha (CD140A), STRO-1, and activated leukocyte cell adhesion molecule (CD166). The expressions of a set of MSC-negative markers (i.e., CD34, CD11b, CD19, CD45, and HLA-DR) and c-Kit (CD117) was also assessed. Although CFs shared some characteristics with MSCs, they presented a specific profile in which MSC markers such as CD140A and STRO-1 were not detected (**Fig 1C**).

Similarly, the expression of CSC markers (i.e., ATP-binding cassette subfamily G member 2 (CD338), NK2 homeobox 5 (NKX2.5), ISL LIM homeobox 1 (Isl1), and GATA-binding protein 4 (GATA4)) was evaluated. CFs are also positive for most of the CSC markers but not for CD338. CFs also shared characteristics with CSCs, but the lack of expression of critical CSC markers such as CD338 in CFs distinguished the two lineages. Additionally, the expression of several markers of EPDCs (i.e., CD46, WT1 transcription factor (WT1), T-box 18 (TBX18), and myocyte enhancer factor 2C (MEF2C)) was evaluated, and CFs presented an EPDC-like profile. CFs also expressed some endothelial markers such as intercellular adhesion molecule 1 (CD54), but other endothelial markers were expressed over various patterns: platelet and endothelial cell adhesion molecule 1 (CD31) was detected over a broad intensity range, and VE-cadherin (CD144) was not detected (**Fig 1C**).

Overall, the absence of various MSC-specific markers confirms that these fibroblasts are not similar to MSCs, and the detection of numerous cardiac-specific markers confirms that these cells have a cardiac origin. Moreover, the expression profile of VCAM1 defines these cells as heterogeneous. Although all lineage-specific markers followed a unimodal distribution in the cell population, VCAM1 showed a clear bimodal distribution, enabling the distinction of two subpopulations: VCFs and VNCFs.

### VCFs have potential roles in triggering blood and lymphatic vessel formation

**Fig 2:**
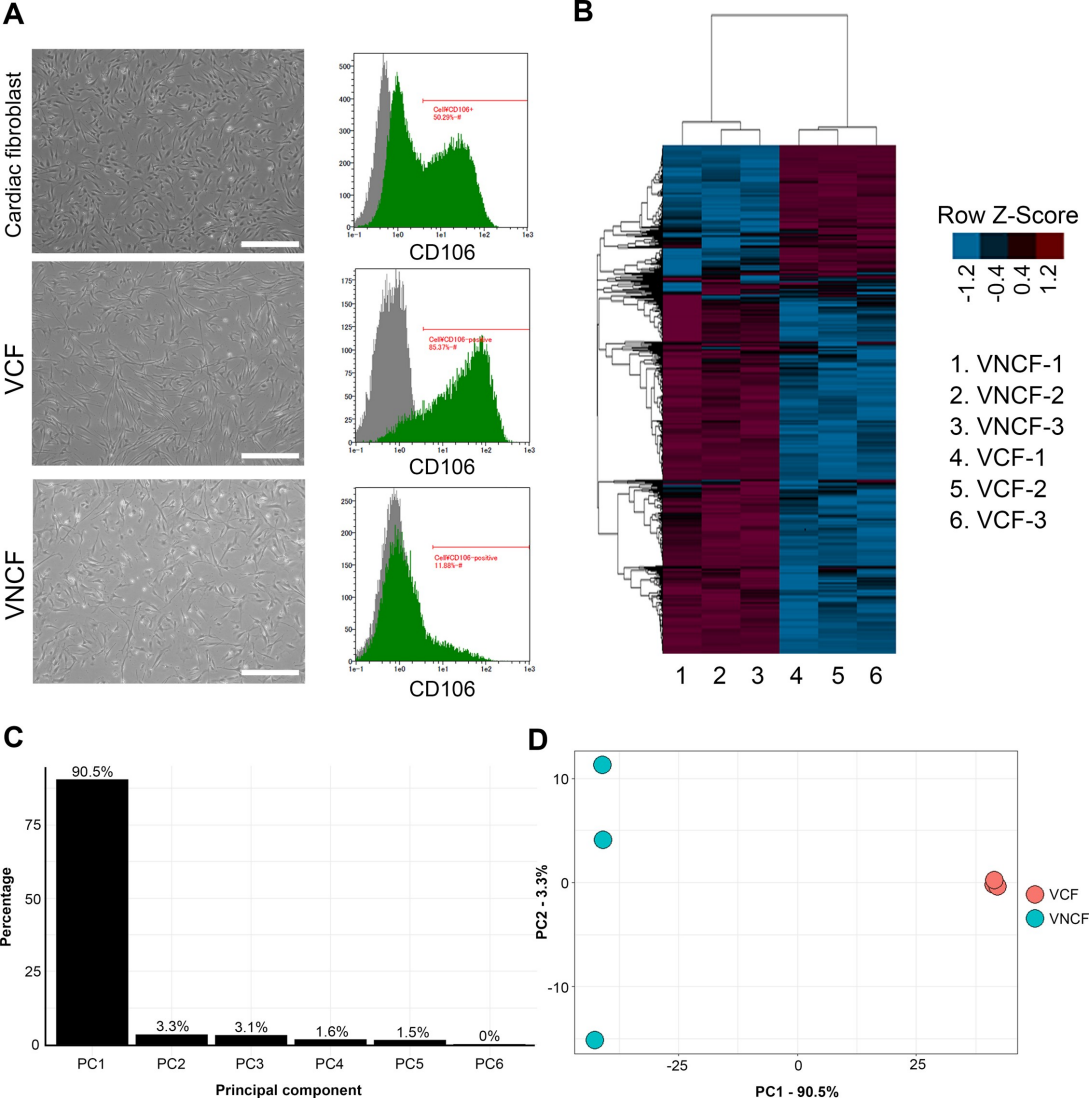
The functional role of differences between VCFs and VNCFs in blood and lymph vessel formation. **A,** Micrographs of CFs, VCFs, and VNCFs cultured for five days, and corresponding immunoprofiling for VCAM1. Gray peaks correspond to the expression pattern of isotype control. **B,** Heatmap of transcriptome showing the specific profiles of VCFs and VNCFs. **C–D,** Scree plot and PC1-2 plot of genes differentially expressed in VCFs and VNCFs showing a clear clustering of both cell types along PC1.

To examine whether the CFs expressing or not VCAM1 have different influences on cardiac function, the transcriptome profile of each population was analyzed by RNA sequencing (n = 3). Two populations were isolated by MACS according to the differential expression of VCAM1, and the purity of VCFs and VNCFs was confirmed by FACS (**Fig 2A**). Whole-transcriptome analysis confirmed that VCFs and VNCFs have substantially distinct profiles, with 2286 genes presenting significantly different expression (|log_2_FC| ≥ 1 and adjusted p ≤ 0.05; **Fig 2B**). Genes differentially expressed in the two populations were ranked by principal component analysis (PCA; **Fig 2C and 2D**) according to their influence on PC1 (i.e., loading score). Each loading score was expressed as a percentage of the most important one, and the last percentile was selected for further investigation (504 candidate genes). This new list was matched to the gene sets “cardiovascular system development” (Gene Ontology 0072358) and “heart failure” (Comparative Toxicogenomic Database D006333). After filtration, 37 candidate genes remained (**S2 Table**). Notably, VCAM1 was ranked 108^th^ in the last percentile list but was not part of GO:0072358 (cardiovascular system development). In the filtered list, 13 genes were upregulated in VCFs [i.e., MYLK, THY1, NR2F2, NRP1, PDGFRB, EGR1, TBX3, CAV1, JUN, Vascular endothelial growth factor C (VEGFC), PTK2B, RAMP1, and FLT1]. We manually searched bibliographical databases and confirmed that all these genes are related to angiogenesis, vascular inflammation/permeability or vascular contraction (i.e., MYLK [29], THY1 [30], NR2F2 [31], NRP1 [32–34], PDGFRB [35], EGR1 [36], TBX3 [37], CAV1 [38], JUN [39], VEGFC [40], PTK2B [41], RAMP1 [42], and FLT1 [33]), and lymphangiogenesis (i.e., THY1 [43], NR2F2 [31], NRP1 [32,34], PDGFRB [44,45], CAV1 [46], VEGFC [19,20,47], RAMP1 [42], and FLT1 [48]). Conversely, many of the genes expressed at higher levels in VNCFs were linked to the ECM-producing myofibroblast phenotype, to the profibrotic signaling (i.e., ACTA2 (alpha smooth muscle actin) [49], CCL2 [50], collagens (COL5A1, COL4A1 and COL4A2) [51], CXCL8 [52], HES1 [53]), to the endothelial mesenchymal transition (EMT) induction (i.e., markers of cardiac EMT ALDH1A2 [54], ANGPTL4 [55], EDN1 [56], WT1 [54], CDH2 (N-cadherin) [57,58]), and to the TGF-β signaling (i.e., ITGAV [59], TGFBI [60], KLF5 [61,62], TGFBR2 [60], PTGS2 [63]). However, inhibitory SMADs (SMAD6/7) regulating the TGF-β superfamily signaling were also expressed at higher levels in VNCFs [54]. Overall, bioinformatics analysis suggests that VCFs express paracrine factors that potentially trigger blood and/or lymph vessel formation.

**Fig 3:**
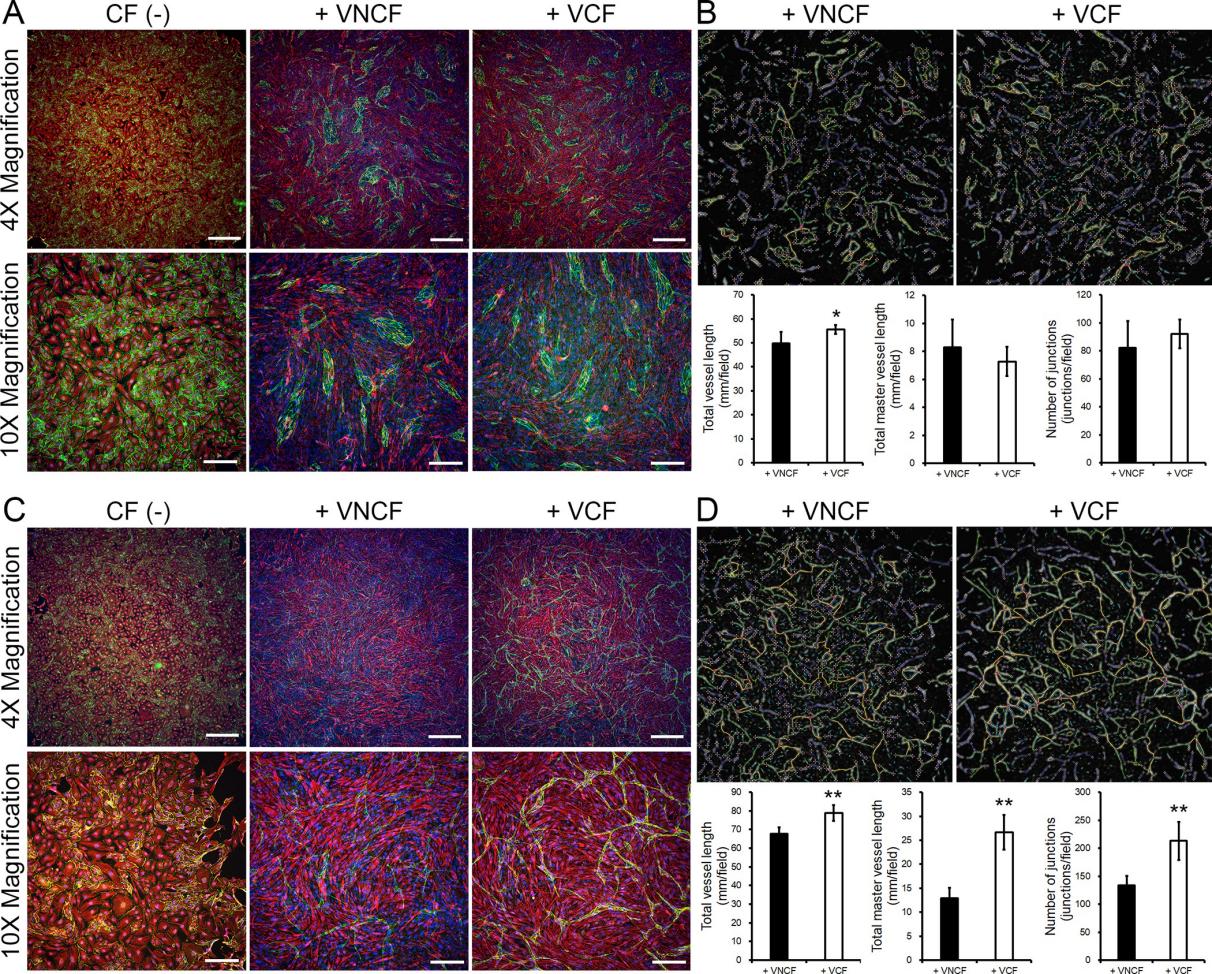
Evaluation of the capacity of VCFs and VNCFs to induce angiogenesis or lymphangiogenesis by tube formation assay. **A,** Micrographs of cultures of HUVECs only (CF (-)) and co-cultures of HUVECs/V(N)CFs (n = 6 per condition; staining: VE-cadherin (green), vimentin (red), and Hoechst 33258 (blue); scale bars: 500 μm (4X) and 200 μm (10X)). **B,** Visualizations of the analysis of tube topography (upper panel) and plots of critical parameters (*t*-test, p < 0.05 (*) versus + VNCF; lower panel). **C,** Micrographs of cultures of HMVEC-Cs only (CF (-)) and co-cultures of HMVEC-Cs/V(N)CFs (n = 5 per condition; staining: VE-cadherin (green), vimentin (red), and Hoechst 33258 (blue); scale bars: 500 μm (4X) and 200 μm (10X)). **D,** Visualizations of the analysis of tube topography (upper panel) and plots of critical parameters (*t*-test, p < 0.01 (**) versus + VNCF; lower panel).

To define the capacity of VCFs to induce angiogenesis or lymphangiogenesis, we assessed endothelial or lymph endothelial vascular network formation. Endothelial cells (i.e., HUVECs) do not form any tubes in standard culture (**Fig 3A**, CF(-)). However, some structures appear in co-cultures with V(N)CFs (**Fig 3A, 3B**). The co-culture of HUVEC with VCFs leads to the formation of significantly longer tubes than with VNCFs (**Fig 3B**, total vessel length). Other network characteristics are comparable in both culture conditions. Similarly to HUVECs, HMVEC-Cs (comprising almost exclusively cardiac lymph endothelial cells as confirmed by FACS against podoplanin and VE-cadherin, **S1 Fig**) do not spontaneously form any tubes (**Fig 3C**, CF (-)). However, these cells react strongly to the presence of VCFs to form complex networks. In these conditions, the co-culture of HMVEC-Cs with VCFs leads to the formation of significantly longer tubes than with VNCFs. Moreover, other network characteristics are also significantly increased by VCFs compared to VNCFs. Finally, the complexity of the network, represented by the length of master vessels and the number of junctions, is highly superior in co-cultures of HMVEC-Cs/VCFs than in any other tested combinations. This observation suggests that while HUVECs only form isolated tubes, HMVEC-Cs create an intricate network under the influence of VCFS.

### VCFs improve the recovery of myocardial function after surgically induced heart failure

**Fig 4:**
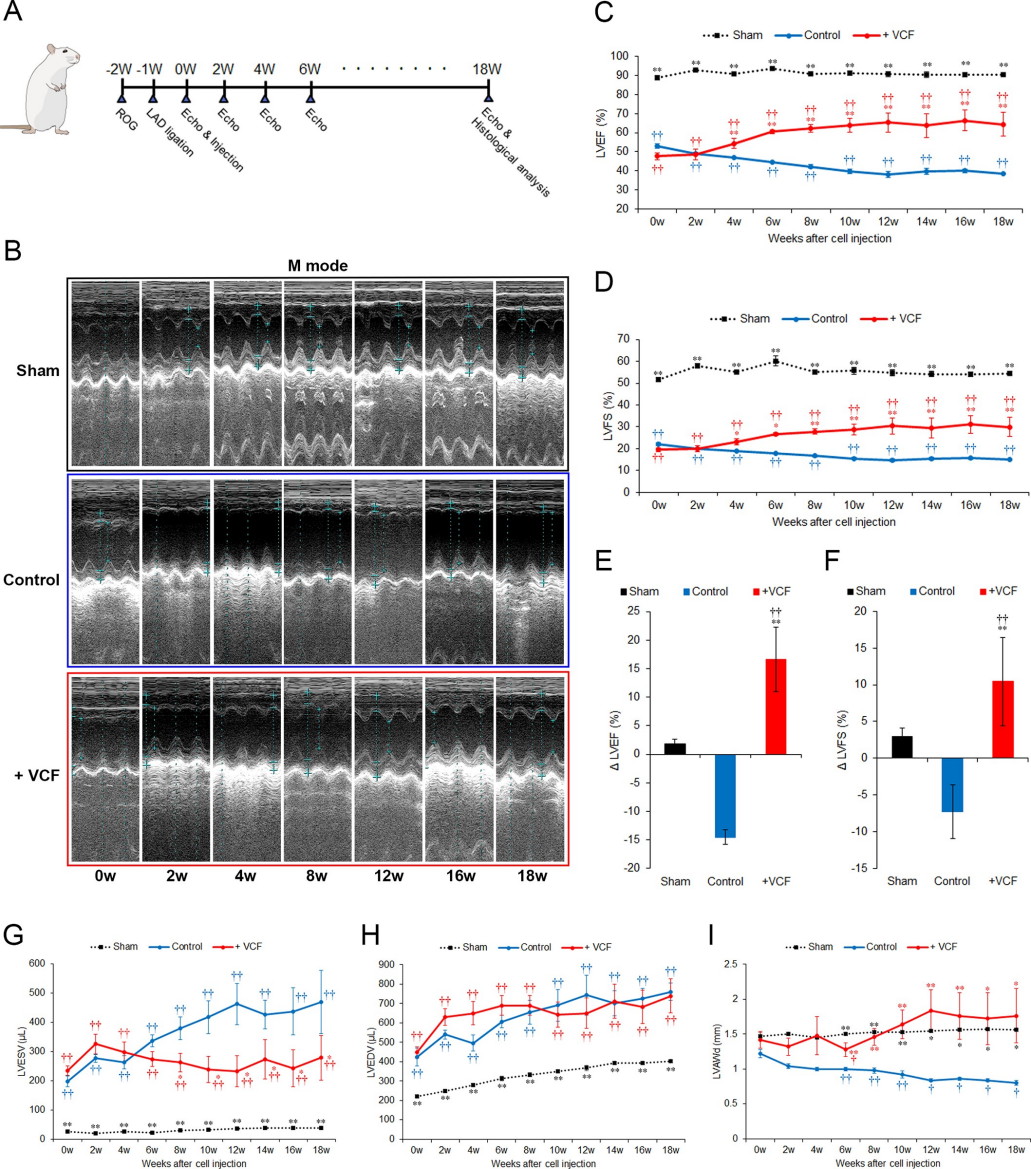
Improvement of cardiac function by VCFs after surgically induced heart failure. **A,** Schedule of surgery and post-surgery operations. VCFs or vehicle were injected into injured tissues one week after infarct. Sham-operated animals had only open-heart surgery without ischemia. Echocardiogram of the left ventricle was performed every 2 weeks over an 18-week period post-injection. At the end of the monitoring period, animals were sacrificed and histological samples were collected. **B,** Representative M-mode echocardiograms of several monitoring points (0 weeks: pre-cell administration). **C–D,** LVEF and LVFS at each monitoring point (mean ± SE, non-parametric Kruskal Wallis test followed by the Dunn post hoc test, p < 0.01 (††) versus sham-treated group, p < 0.01 (**), p < 0.05 (*) versus control group). **E–F,** Delta in LVEF and LVFS values between the end of the experiment (18 weeks) and prior to cell administration (0 weeks) (p < 0.01 (††) versus sham-treated group, p < 0.01 (**) versus control group). **G–I,** LVESV, LVEDV, and LVAWd at each monitoring point (p < 0.01 (††), p < 0.05 (†) versus sham-treated group, p < 0.01 (**), p < 0.05 (*) versus control group).

To assess whether VCFs have the potential to improve cardiac function after heart failure by triggering angiogenesis and/or lymphangiogenesis, heart failure was induced in rats by ligation of the left anterior descending artery. A schematic of the experimental procedure is presented in **Fig 4A**. Echocardiography in rats of the sham-treated group showed that the high amplitude left ventricular anterior wall motion was maintained throughout the whole monitoring period (**Fig 4B**). Conversely, rats of the vehicle-treated control group presented dramatically reduced left ventricular wall motion at all monitoring points, confirming the successful induction of the infarct model, and had a slight further degradation over the course of the experiment (**Fig 4B**). Rats treated with VCFs presented the same initial (pre-cell administration, 0 weeks of treatment) reduction of wall motion as rats in the vehicle-treated control group, but VCF administration led to an improvement in wall motion amplitude less than 1 month after treatment (**Fig 4B**, **S2 Fig**).

Consistent with the improvement of anterior wall motion, VCF-treated rats presented a substantial increase in LVEF and LVFS. Sham-treated rats presented an LVEF and LVFS of approximately 90% of 55% respectively. After infarct and before the injections, LVEF and LVFS were drastically reduced to 50% and 20%, respectively, in both the vehicle- and VCF-treated groups. Although improvement was observed in the VCF-treated group at the first monitoring point (2 weeks post-injection), a significant difference between the vehicle- and VCF-treated groups emerged by 4 weeks of treatment. From roughly 10 weeks after injection onward, the LVEF and LVFS plateaued in both groups. The final LVEF values 18 weeks after injection were 64.35% ± 6.15% in VCF-treated rats and 38.40% ± 0.41% in vehicle-treated control animals. The final LVFS values were 29.90% ± 4.35% in the VCF-treated group and 14.90% ± 0.20% in the vehicle-treated control group (**Fig 4C and 4D**). Over the course of the experiment, LVEF and LVFS increased by 16.65% ± 5.64% and 10.43% ± 6.02%, respectively, in VCF-treated rats and decreased by 14.53% ± 1.23% and 7.33% ± 3.66% in the vehicle-treated control group (**Fig 4E and 4F**). Although a return to physiological LVEF and LVFS was not achieved in this study, VCF treatment provided a significant improvement over the control from four weeks after injection that improved even further and then was maintained until the end of the experiment.

Left ventricular end-systolic and end-diastolic volumes (LVESV and LVEDV respectively) were measured in all groups. In the sham-treated group, LVESV was stable at less than 50 μl throughout the 18 weeks of monitoring. In the vehicle-treated control group, the LVESV continually increased to 469.00 ± 108.60 μl. VCF treatment limited the increase to 278.75 ± 77.27 μl, with a significant effect relative to the vehicle-treated group observed eight weeks after injection. However, all groups showed the same evolution of LVEDV throughout the experiment (**Fig 4G and 4H**). Finally, VCF treatment improved the left ventricular anterior wall thickness in diastole (LVAWd) to 1.76 ± 0.39 mm, similar to that in the sham-treated group (1.56 ± 0.02 mm). In contrast, the LVAWd was continuously degraded in vehicle-treated control animals (0.80 ± 0.03, **Fig 4I**). Values and statistical analyses of all echocardiographic measurements are detailed in **S3 Table**. VCFs thus appear to improve heart function by enhancing ventricular wall contractility.

### VCFs stimulate the mobilization of lymphatic endothelial cells in the infarct zone

The tissue remodeling capacity of VCFs was assessed by histological analysis of hearts collected after 18 weeks of treatment. H&E and SR staining were performed on transverse sections of ventricles.

**Fig 5:**
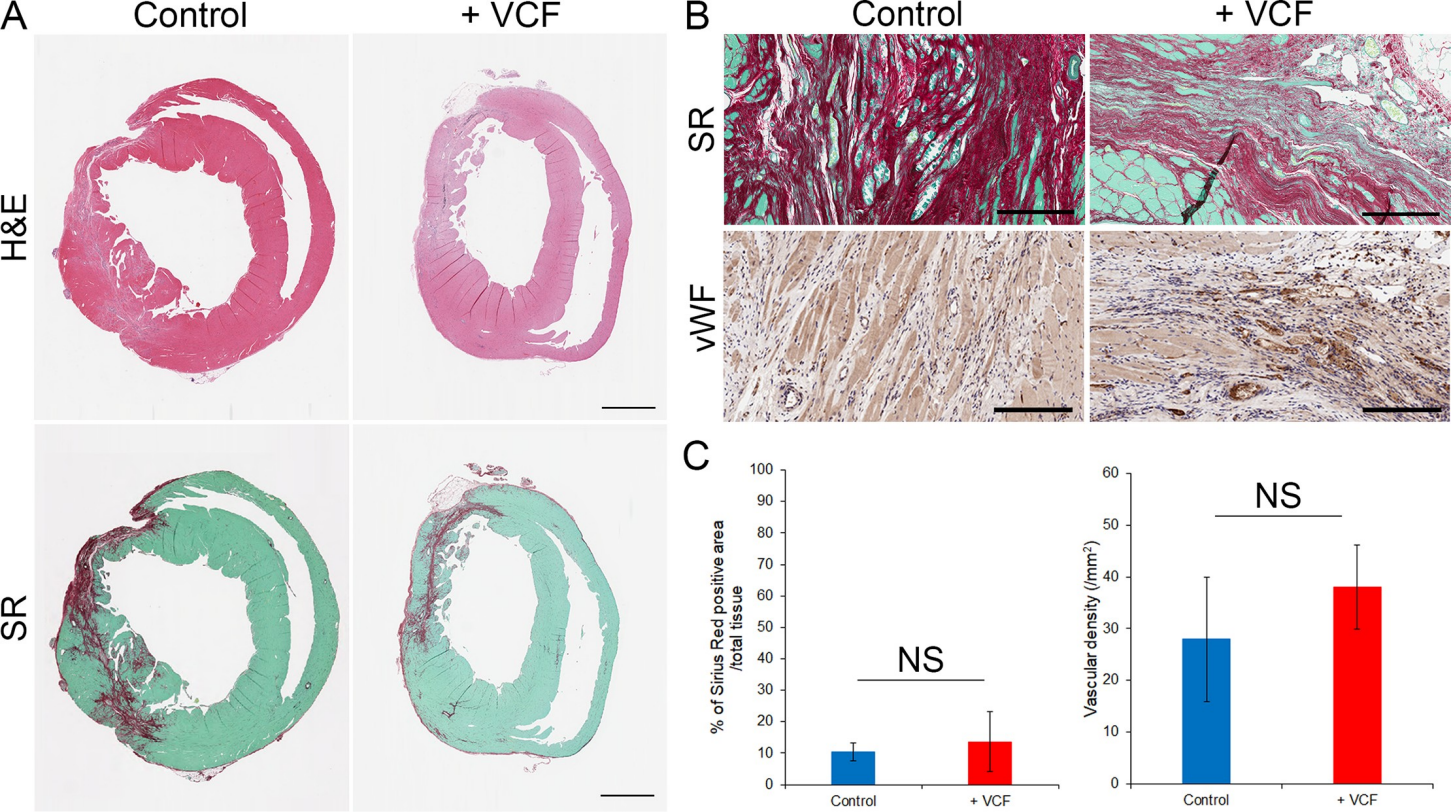
Effect of VCF treatment on fibrosis and angiogenesis in infarcted tissues. **A,** Hematoxylin and eosin (H&E) and Sirius Red (SR) staining of heart sections 18 weeks post-injection (scale bars: 2 mm). **B,** Micrographs of injured regions in SR stained or vWF stained heart sections (scale bars: 200 μm). **C,** Quantification of fibrosis extent and blood vessel density in control and VCF-treated groups (NS: no significant difference).

Microscopic observation revealed similar histological structures in control and VCF-treated groups. SR staining revealed the presence of fibrotic structures in the infarcted tissues of both groups (**Fig 5A**). The extent of fibrosis (i.e., the ratio of the surface area of SR-stained tissue to the whole surface area of the section) was not significantly different between the VCF- and vehicle-treated groups (**Fig 5B**). The fibrosis ratio was 10.49 ± 1.21% and 13.57 ± 4.84% in the vehicle- and VCF-treated groups, respectively (**Fig 5C**). Additionally, the immunostaining of von Willebrand Factor (vWF) for estimation of the density of blood vessels in the fibrotic area (number of vessels per mm^2^) suggested a slight increase in the number of blood vessels in infarcts of VCF-treated rats, but did not show significant difference between the two groups (**Fig 5B**). The vascular density was 27.93 ± 11.97 vessels/mm^2^ and 38.07 ± 8.12 vessels/mm^2^ in the vehicle- and VCF-treated groups, respectively (**Fig 5C**).

**Fig 6:**
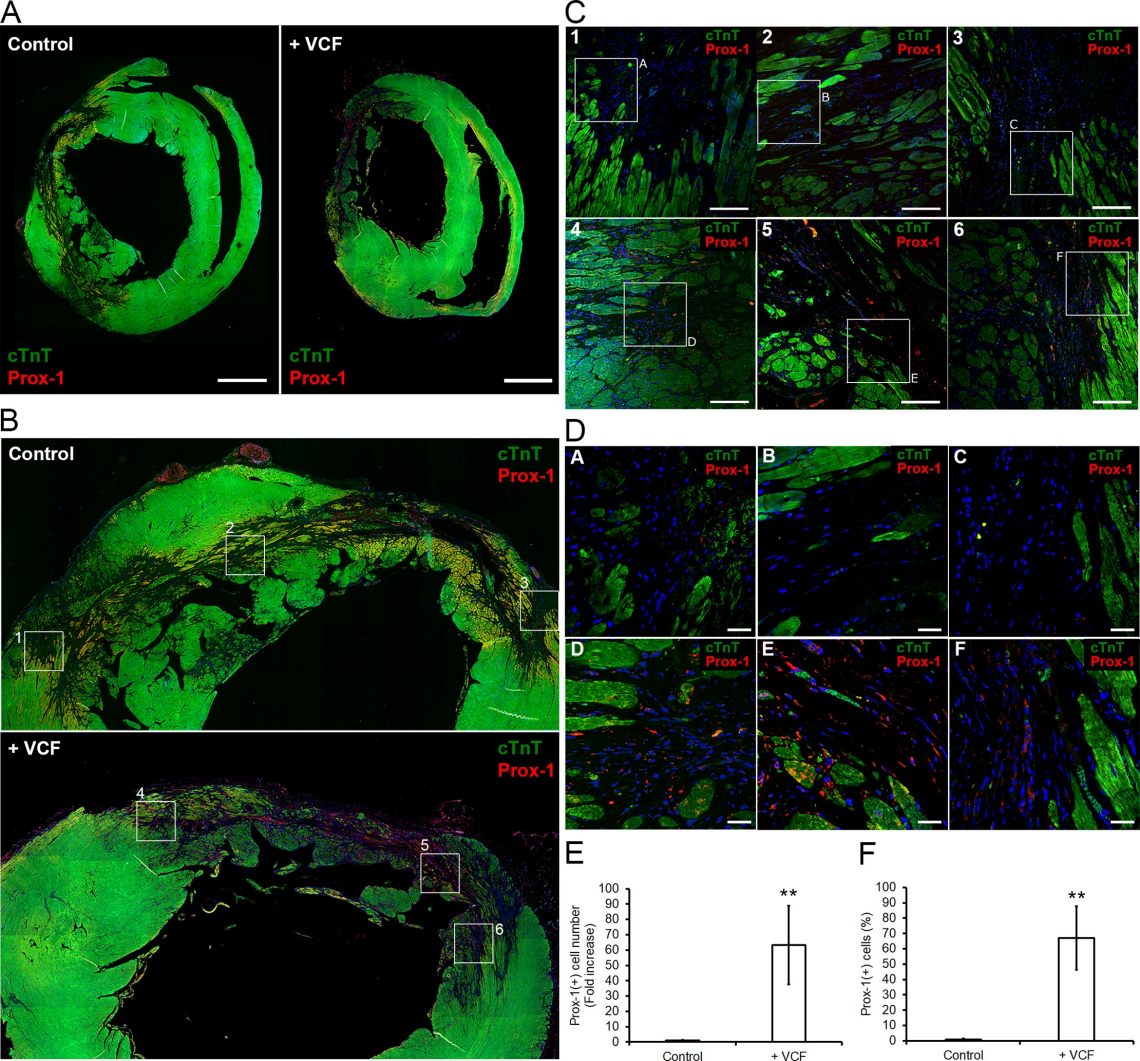
Effect of VCFs on the lymphatic vasculature of the infarct. **A,** Localization of lymphatic endothelial cells (LECs) in hearts of vehicle control (left) and VCF-treated groups (right): cTnT (green), Prox1 (red), and nucleus (blue) (scale bars: 2 mm). **B,** Magnification on the infarcted area: cTnT (green), Prox1 (red), nucleus (blue). **C,** Magnification of areas indicated on B. Micrographs 4, 5, and 6 (from VCF-treated animals) show the important migration of LECs into the infarcted area compared to micrographs 1, 2, and 3 (from vehicle-treated animals) (scale bars: 500 μm). **D,** Magnification of areas indicated on C. Micrographs D, E, and F (from VCF-treated animals) show the important migration of LECs into the infarcted area compared to micrographs A, B, and C (from vehicle-treated animals) (scale bars: 200 μm). **E,** Number of LECs in injured regions of each group. Bar graphs depict the fold increase in Prox-1(+) cells in the infarcts. The value for vehicle-treated animals was set as 1.0 (n = 4, p < 0.01 (**)). **F,** The percentage of mobilized-LECs in the infarcts expressing Prox-1. The value for vehicle-treated animals was set as 1.0 (n = 4, p < 0.01 (**)).

Thus, to test whether the dramatic improvement of mechanical properties and function was caused by the lymphangiogenesis effects of VCFs as suggested by the transcriptome analysis and lymph vessel formation assays (**Figs 2 and 3**), we performed immunostaining of cTnT and Prox-1 in heart sections. Prox-1 is a transcription factor specific to lymphatic endothelial cells (LECs) that is necessary for lymphangiogenesis and the maintenance of lymphatic vessels [64]. VCF-treated groups showed enrichment of LECs in and around infarcted tissues (**Fig 6A and 6B**). Higher-magnification confocal microscopic images showed that VCFs appeared to efficiently mobilize LECs (**Fig 6C and 6D**). Imaging analysis was performed to assess the mobilization of LECs in control or VCF-treated infarcts. VCF treatment significantly promoted the mobilization of LECs by 63.18 ± 25.61 times (number of Prox-1(+) cells) compared to the control medium injection alone. Moreover, 67.10 ± 20.67% of the cells recruited to the infarcts in the VCF-treatment groups were LECs. These findings suggest that VCFs restore cardiac function after heart failure following myocardial infarction by inducing lymphangiogenesis.

## Discussion

CFs have recently been shown to maintain proper heart function through multiple interactions with resident cells of the myocardium even after cardiac injury and cardiac diseases [6,8–10]. However, in pathological states, CFs are also the principal determinants of cardiac fibrosis and eventually lead to cardiac remodeling and heart failure [14,15]. Thus, CFs show completely different characteristics depending on the conditions of the heart tissue environment. The factors and mechanisms regulating normal heart function have yet to be described precisely. In this study, we hypothesized that CFs are a heterogeneous population of cells, and we demonstrated that a specific population of CFs expressing VCAM1 presented an up-regulation of blood and/or lymph vessel formation-related genes compared with CFs not expressing VCAM1. Vascular formation assays showed VCFs are more efficient at stimulating lymphangiogenesis than angiogenesis. Therefore, we focused on the lymph vessel formation ability of VCFs and showed their ability to restore cardiac function on heart failure following myocardial infarction by triggering lymphangiogenesis.

Several recent studies have suggested that CFs are a heterogeneous cell population and that such heterogeneity is caused by the distinct origins of those cells. CFs originate from resident fibroblasts, from bone marrow-derived progenitor cells (MSCs), from CSCs, from EPDCs via an epithelial-to-mesenchymal transition, or from endothelial cells via an endothelial-to-mesenchymal transition [10,17,65]. In this study, we performed high-throughput cell profiling for the co-expression of CD90 and various CF lineage markers to determine the heterogeneous characteristics of human CFs. CFs shared some characteristics with their originating cell type (e.g., MSCs, CSCs, EPDCs, and endothelial cells). Interestingly, we found that two populations of CFs could be distinguished based on their expression of VCAM1 (**Fig 1C**). VCAM1 is the ligand for α4β1 integrin, which is known to exist as both a transmembrane protein and in a soluble form. Several groups, including ours, have reported that VCAM1 is expressed in cardiomyocytes [66], CSCs [2], and CFs [67]. Soluble VCAM1 secreted from murine CSCs demonstrated cardioprotective effects in mouse through the α4β1 integrin-mediated activation of Akt, ERK, and P38MAPK in cardiomyocytes [2]; this phenomenon has also been reported to be critical for heart development [68,69]. Another study showed that mouse CFs induced the proliferation of mouse ESC-derived cardiomyocytes via the two forms of VCAM1, which led to an increase in functionality of bioengineered myocardial tissues [67]. Therefore, we hypothesized that VCAM1 from human CFs might be involved in heart protection and cardiac cell proliferation. However, our bioinformatics analysis of RNA sequencing unexpectedly suggested that CFs expressing VCAM1 (VCFs) might have an effect on the positive regulation of blood and/or lymph vessel formation (**S2 Table**). Additionally, vessel formation assays revealed that VCFs significantly improved the length of vessels and the complexity of the network of lymphatic endothelial cells (**Fig 3**).

These results suggested the new hypothesis that VCFs enhance heart function in postinfarct heart failure by regulating lymphangiogenesis.

Angiogenesis is a complex blood vessel formation process. Numerous studies have reported that therapeutic angiogenesis, which improves blood flow, revascularization, and myocardial function, is one of the most promising treatments for cardiovascular disease [1]. Lymphangiogenesis is also an important process for the maintenance of normal organ homeostasis and wound healing. Cardiac lymphangiogenesis plays a central role in maintaining fluid homeostasis for healthy heart contraction [18]. Recent studies have suggested that lymphangiogenesis improves cardiac function and healing by draining accumulated fluids and maintaining the physiological interstitial fluid equilibrium [19,20,70]. These studies, together with our RNA sequencing data, suggested that VCFs have a favorable expression of genes related to the regulation of blood and/or lymph vessel formation. For example, vascular endothelial growth factor receptor 1 (FLT1) and neuropilin 1 (NRP1) are VEGF receptors or co-receptors, that act as modulators of VEGF-dependent signaling for angiogenesis or lymphangiogenesis [32–34]. Additionally, the PDGF/PDGFR axis is known as an important regulator of blood vessel development and lymphangiogenesis [44,45]. VEGF-C has been identified as a key molecule in cardiac angiogenesis and lymphangiogenesis through the activation of the VEGFC/VEGFR-3 pathway, leading to the improvement of cardiac function after myocardial infarction [19,20,47]. Conversely, VNCFs express genes promoting cardiac remodeling. Indeed, genes up-regulated in VNCFs compared to VCFs were involved in ECM-producing myofibroblast phenotypes, profibrotic signaling, and EMT induction. In particular, ACTA2 [49], TGF-β signaling related proteins (ITGAV [59], TGFBI [60], KLF5 [61,62], TGFBR2 [60], and PTGS2 [63]), and markers of cardiac EMT (ALDH1A2 [54], ANGPTL4 [55], EDN1 [56], WT1 [54], and CDH2 (N-cadherin) [57,58]) are well-known as profibrotic factors [55,62,71,72]. EDN1 is a protein that CFs accumulate through cardiac EMT in diabetic hearts [73]. It also promotes collagen synthesis in the senescent fibroblasts and promotes cardiac EMT, leading to myocardial fibrosis [74]. ITGAV, expressed by fibroblasts, is a well-known key regulator of fibrogenesis that acts by activating TGF-β signaling through its interaction with a linear arginine-glycine-aspartic acid-binding motif that is present on the latency-associated peptide in ECM [59]. Additionally, SMADs (SMAD6 and 7), inhibitors of the TGF-β superfamily signaling [54], were also more expressed in in VNCFs than in VCFs. Although we did not interpret these conflicting results, VNCFs might play a role in regulating ECM and cardiac EMT by responding to the status of the *in vitro/in vivo* environment. Moreover, endothelial/lymph endothelial vessel formation assays showed the capacity of VCFs and VNCFs to induce the creation of lymphatic tube networks. These data support the new hypothesis that VCFs, by enhancing lymphatic vessel formation, may restore cardiac function in heart failure following myocardial infarction.

Indeed, we found that injected VCFs led to a significant improvement in heart contractile function in rats after surgically induced heart failure. Over the course of the experiment (18 weeks after injection), VCF administration produced a definite improvement in left ventricular anterior wall motion, LVEF, and LVFS (**Fig 4B–4F**). The necrosis following infarction causes a decrease of the thickness of the ventricular wall, but VCFs treatment maintained this parameter close to physiological values (**Fig 4I**), suggesting an improved protection of injured tissues. Similar to the in vitro vessel formation assay (**Fig 3**), this favorable effect was not induced by angiogenesis (**Fig 5**) but by lymphangiogenesis (**Fig 6**).

Lymphangiogenesis attenuates inflammatory disorders and cardiac fibrosis by inhibiting the swelling and the formation of interstitial edema following muscular necrosis [18,75,76]. In this study, VCF treatment appeared to efficiently mobilize LECs in the infarct zone (**Fig 6**), but there was no significant difference between the VCF and control groups in the extent of fibrosis (**Fig 5**). Similarly, Trincot et al. reported that adrenomedullin overexpression induces mouse cardiac lymphangiogenesis after myocardial infarction, but the area of fibrosis is not changed [70]. Although further studies are necessary to understand the relations between lymphangiogenesis and fibrosis, regulatory T cells seems involved as they have been shown to improve cardiac function after myocardial infarction via the inhibition of inflammation and the attenuation of interstitial fibrosis [16,77]. We suspect that the maintenance of immune balance by VCF-induced lymphangiogenesis might have been insufficient because we used F344/N Jcl-rnu/rnu rats immunologically lacking T-cell functions for the animal experiments. Another possibility is that paracrine factors from VCFs might be insufficient for reducing fibrosis resulting from long-term inflammation, although VCFs can improve heart contractile function.

This study has a few limitations. We used cells isolated from human fetal hearts as the CFs. Although almost all of these cells resembled fibroblasts morphologically and were positive for fibroblast markers, we cannot exclude the possibility that these populations also contained other cell types. Additionally, 2286 genes were differentially expressed between in VCFs and VNCFs; thus, it is reasonable to assume the contribution of pathways other than angiogenesis and lymphangiogenesis such as cardiac protection or cardiac proliferation. Additionally, the analysis of vessel formation in vitro do not represent perfectly the structure of the network. In this experiment, angiogenesis analysis was performed on a 2D projection (1 micrograph) of a 3D microtissue (co-culture); thus, part of the volume information is lost, which led to segmentation of the network. Moreover, because the lymphangiogenic effect was evaluated by the same method as previously reported for angiogenesis [21], the protocol may be sub-optimized. Finally, no immune cells were integrated into the in vitro experiments presented here. Immune cells such as macrophages contribute to the process of lymphatic remodeling by stimulating lymphangiogenesis [78]. In health, lung injury leads to the activation of CD11b^+^ macrophages, which transdifferentiate into LECs [79–81].

Moreover, the mechanisms of lymphatic endothelial cell-fibroblast signaling have not yet been elucidated. As mentioned above, VEGFC, which is listed in **S2 Table** as a gene upregulated in VCFs, is involved in cardiac lymphangiogenesis through the activation of the VEGFC/VEGFR-3 pathway leading to the improvement of cardiac function after myocardial infarction [19,20,47]. However, it is unclear whether VCFs have the potential to improve the signal transduction of the VEGFC/VEGFR-3 axis; therefore, rapamycin (therapeutically available as sirolimus)-treated rats should be investigated to confirm the involvement of the signaling pathways. Rapamycin inhibits the expression of VEGFC and significantly suppresses tumor-related lymphangiogenesis and lymph node metastasis in mammals [81]. Finally, it remains elusive whether VCFs are also present in the human adult heart, and whether they provide similar effects on heart failure.

In summary, these results demonstrate that a specific population of cultured human fetal heart-derived fibroblasts expressing VCAM1 has a role in lymphangiogenesis, and that a treatment with VCFs restores cardiac contractile functions on heart failure following myocardial infarction by mobilizing lymph endothelial cells into the infarct. Understanding the molecular mechanism mediating lymphangiogenesis may provide new insights in cardiac development and pathogenesis, and lead to new options in the field of heart regeneration. Our next challenge is to demonstrate these effects with human adult heart-derived fibroblasts and identify the molecular signaling mechanisms that trigger lymphangiogenesis after treatment with VCFs.

## Supporting information

S1 Table Antibodies and reagents used for MACS, FACS, vascular formation assay, and immunohistochemistry. (DOCX)

S2 Table List of genes differentially expressed in VCFs compared to VNCFs (limited to the top 1% of loading scores) that are referenced in “cardiovascular system development” and in “heart failure” gene sets. The list is sorted in descending order of the loading scores as listed in the PCA output.
(DOCX)

S3 Table Echocardiography values and corresponding statistical analysis. (DOCX)

S1 Fig Flowcytometry analysis of HMVEC-Cs. Markers of lymphatic endothelial cells are detected in a large majority of HMVEC-Cs. Gray peaks correspond to the expression pattern of isotype control.
(DOCX)

S2 Fig Representative echocardiographic images at each monitoring point (0 weeks: Pre-cell administration). In each M-mode image, the long light blue dotted line shows the measurement position at the diastolic phase, and the short light blue dotted line shows the measurement position at the systolic phase.
(DOCX)

S1 Text Whole transcriptome analysis. (DOCX)

S2 Text Animal experiments. (DOCX)

## References

[1] Sánchez-Alonso S, Alcaraz-Serna A, Sánchez-Madrid F, Alfranca A. Extracellular Vesicle-Mediated Immune Regulation of Tissue Remodeling and Angiogenesis After Myocardial Infarction. Front Immunol. 2018;9: 2799 10.3389/fimmu.2018.02799 30555478 PMC6281951

[2] Matsuura K, Honda A, Nagai T, Fukushima N, Iwanaga K, Tokunaga M, et al Transplantation of cardiac progenitor cells ameliorates cardiac dysfunction after myocardial infarction in mice. J Clin Invest. 2009;119: 2204–2217. 10.1172/JCI37456 19620770 PMC2719947

[3] Liu YW, Chen B, Yang X, Fugate JA, Kalucki FA, Futakuchi-Tsuchida A, et al Human embryonic stem cell-derived cardiomyocytes restore function in infarcted hearts of non-human primates. Nat Biotechnol. 2018;36: 597–605. 10.1038/nbt.4162 29969440 PMC6329375

[4] Werner JH, Rosenberg JH, Um JY, Moulton MJ, Agrawal DK. Molecular discoveries and treatment strategies by direct reprogramming in cardiac regeneration. Transl Res. 2019;203: 73–87. 10.1016/j.trsl.2018.07.012 30142308 PMC6289806

[5] Breckwoldt K, Weinberger F, Eschenhagen T. Heart regeneration. Biochim Biophys Acta—Mol Cell Res. 2016;1863: 1749–1759. 10.1016/j.bbamcr.2015.11.010 26597703

[6] Porter KE, Turner NA. Cardiac fibroblasts: At the heart of myocardial remodeling. Pharmacol Ther. 2009;123: 255–278. 10.1016/j.pharmthera.2009.05.002 19460403

[7] Jimenez-Tellez N, Greenway SC. Cellular models for human cardiomyopathy: What is the best option? World J Cardiol. 2019;11: 221–235. 10.4330/wjc.v11.i10.221 31754410 PMC6859298

[8] Díaz-Araya G, Vivar R, Humeres C, Boza P, Bolivar S, Muñoz C. Cardiac fibroblasts as sentinel cells in cardiac tissue: Receptors, signaling pathways and cellular functions. Pharmacol Res. 2015;101: 30–40. 10.1016/j.phrs.2015.07.001 26151416

[9] Deschamps AM, Spinale FG. Disruptions and detours in the myocardial matrix highway and heart failure. Curr Heart Fail Rep. 2005;2: 10–17. 10.1007/s11897-005-0002-6 16036046

[10] Souders CA, Bowers SLK, Baudino TA. Cardiac Fibroblast. Circ Res. 2009;105: 1164–1176. 10.1161/CIRCRESAHA.109.209809 19959782 PMC3345531

[11] Zamani M, Karaca E, Huang NF. Multicellular Interactions in 3D Engineered Myocardial Tissue. Front Cardiovasc Med. 2018;5: 147 10.3389/fcvm.2018.00147 30406114 PMC6205951

[12] Matsuura K, Masuda S, Haraguchi Y, Yasuda N, Shimizu T, Hagiwara N, et al Creation of mouse embryonic stem cell-derived cardiac cell sheets. Biomaterials. 2011;32: 7355–7362. 10.1016/j.biomaterials.2011.05.042 21807408

[13] Iseoka H, Miyagawa S, Fukushima S, Saito A, Masuda S, Yajima S, et al Pivotal Role of Non-cardiomyocytes in Electromechanical and Therapeutic Potential of Induced Pluripotent Stem Cell-Derived Engineered Cardiac Tissue. Tissue Eng Part A. 2017;24: ten.tea.2016.0535. 10.1089/ten.tea.2016.0535 28498040 PMC5792250

[14] Wynn TA. Common and unique mechanisms regulate fibrosis in various fibroproliferative diseases. J Clin Invest. 2007;117: 524–529. 10.1172/JCI31487 17332879 PMC1804380

[15] Fujisaki H, Ito H, Hirata Y, Tanaka M, Hata M, Lin M, et al Natriuretic peptides inhibit angiotensin II-induced proliferation of rat cardiac fibroblasts by blocking endothelin-1 gene expression. J Clin Invest. 1995;96: 1059–1065. 10.1172/JCI118092 7635942 PMC185295

[16] Blanton RM, Carrillo-Salinas FJ, Alcaide P. T-cell recruitment to the heart: friendly guests or unwelcome visitors? Am J Physiol Circ Physiol. 2019;317: H124–H140. 10.1152/ajpheart.00028.2019 31074651 PMC6692732

[17] Furtado MB, Costa MW, Pranoto EA, Salimova E, Pinto AR, Lam NT, et al Cardiogenic genes expressed in cardiac fibroblasts contribute to heart development and repair. Circ Res. 2014;114: 1422–1434. 10.1161/CIRCRESAHA.114.302530 24650916 PMC4083003

[18] Huang LH, Lavine KJ, Randolph GJ. Cardiac Lymphatic Vessels, Transport, and Healing of the Infarcted Heart. JACC Basic to Transl Sci. 2017;2: 477–483. 10.1016/j.jacbts.2017.02.005 28989985 PMC5628514

[19] Henri O, Pouehe C, Houssari M, Galas L, Nicol L, Edwards-Lévy F, et al Selective Stimulation of Cardiac Lymphangiogenesis Reduces Myocardial Edema and Fibrosis Leading to Improved Cardiac Function Following Myocardial Infarction. Circulation. 2016;133: 1484–1497. 10.1161/CIRCULATIONAHA.115.020143 26933083

[20] Shimizu Y, Polavarapu R, Eskla KL, Pantner Y, Nicholson CK, Ishii M, et al Impact of lymphangiogenesis on cardiac remodeling after ischemia and reperfusion injury. J Am Heart Assoc. 2018;7: 1–14. 10.1161/JAHA.118.009565 30371303 PMC6404883

[21] Masuda S, Matsuura K, Shimizu T. Inhibition of LYPD1 is critical for endothelial network formation in bioengineered tissue with human cardiac fibroblasts. Biomaterials. 2018;166: 109–121. 10.1016/j.biomaterials.2018.03.002 29550615

[22] Pop S, Dufour A, Olivo-Marin J. Image filtering using anisotropic structure tensor for cell membrane enhancement in 3D microscopy. 2011 18th IEEE International Conference on Image Processing. 2011 pp. 2041–2044. 10.1109/ICIP.2011.6115880

[23] Koga T, Fukazawa M, Suzuki Y, Akima M, Adachi Y, Tamura K, et al The protective effects of CP-060S on ischaemia- and reperfusion- induced arrhythmias in anaesthetized rats. Br J Pharmacol. 1998;123: 1409–1417. 10.1038/sj.bjp.0701742 9579737 PMC1565298

[24] Yu P, Zhang J, Yu S, Luo Z, Hua F, Yuan L, et al Protective Effect of Sevoflurane Postconditioning against Cardiac Ischemia/Reperfusion Injury via Ameliorating Mitochondrial Impairment, Oxidative Stress and Rescuing Autophagic Clearance. PLoS One. 2015;10: e0134666–e0134666. 10.1371/journal.pone.0134666 26263161 PMC4532466

[25] Chen X, Lu M, Ma N, Yin G, Cui C, Zhao S. Dynamic Tracking of Injected Mesenchymal Stem Cells after Myocardial Infarction in Rats: A Serial 7T MRI Study. Stem Cells Int. 2016/08/30. 2016;2016: 4656539 10.1155/2016/4656539 27656215 PMC5021478

[26] Dominici M, Le Blanc K, Mueller I, Slaper-Cortenbach I, Marini F, Krause D, et al Minimal criteria for defining multipotent mesenchymal stromal cells. The International Society for Cellular Therapy position statement. Cytotherapy. 2006;8: 315–317. 10.1080/14653240600855905 16923606

[27] Uezumi A, Fukada S, Yamamoto N, Takeda S, Tsuchida K. Mesenchymal progenitors distinct from satellite cells contribute to ectopic fat cell formation in skeletal muscle. Nat Cell Biol. 2010;12: 143–152. 10.1038/ncb2014 20081842

[28] Simmons PJ, Torok-Storb B. Identification of stromal cell precursors in human bone marrow by a novel monoclonal antibody, STRO-1. Blood. 1991;78: 55–62. 2070060

[29] Christie JD, Ma S-F, Aplenc R, Li M, Lanken PN, Shah C V, et al Variation in the myosin light chain kinase gene is associated with development of acute lung injury after major trauma. Crit Care Med. 2008;36: 2794–2800. 10.1097/ccm.0b013e318186b843 18828194

[30] Kumar A, Bhanja A, Bhattacharyya J, Jaganathan BG. Multiple roles of CD90 in cancer. Tumour Biol. 2016;37: 11611–11622. 10.1007/s13277-016-5112-0 27337957

[31] Pereira FA, Qiu Y, Zhou G, Tsai MJ, Tsai SY. The orphan nuclear receptor COUP-TFII is required for angiogenesis and heart development. Genes Dev. 1999;13: 1037–1049. 10.1101/gad.13.8.1037 10215630 PMC316637

[32] Djordjevic S, Driscoll PC. Targeting VEGF signalling via the neuropilin co-receptor. Drug Discov Today. 2013;18: 447–455. 10.1016/j.drudis.2012.11.013 23228652

[33] Bernatchez PN, Rollin S, Soker S, Sirois MG. Relative effects of VEGF-A and VEGF-C on endothelial cell proliferation, migration and PAF synthesis: Role of neuropilin-1. J Cell Biochem. 2002;85: 629–639. 10.1002/jcb.10155 11968003

[34] Saban MR, Sferra TJ, Davis CA, Simpson C, Allen A, Maier J, et al Neuropilin-VEGF signaling pathway acts as a key modulator of vascular, lymphatic, and inflammatory cell responses of the bladder to intravesical BCG treatment. Am J Physiol—Ren Physiol. 2010;299 10.1152/ajprenal.00352.2010 20861073

[35] Magnusson PU, Looman C, Ahgren A, Wu Y, Claesson-Welsh L, Heuchel RL. Platelet-derived growth factor receptor-beta constitutive activity promotes angiogenesis in vivo and in vitro. Arterioscler Thromb Vasc Biol. 2007;27: 2142–2149. 10.1161/01.ATV.0000282198.60701.94 17656670

[36] Fahmy RG, Dass CR, Sun L-Q, Chesterman CN, Khachigian LM. Transcription factor Egr-1 supports FGF-dependent angiogenesis during neovascularization and tumor growth. Nat Med. 2003;9: 1026–1032. 10.1038/nm905 12872165

[37] Perkhofer L, Walter K, Costa IG, Carrasco MCR, Eiseler T, Hafner S, et al Tbx3 fosters pancreatic cancer growth by increased angiogenesis and activin/nodal-dependent induction of stemness. Stem Cell Res. 2016;17: 367–378. 10.1016/j.scr.2016.08.007 27632063

[38] Tahir SA, Park S, Thompson TC. Caveolin-1 regulates VEGF-stimulated angiogenic activities in prostate cancer and endothelial cells. Cancer Biol Ther. 2009;8: 2286–2296. 10.4161/cbt.8.23.10138 19923922 PMC2887683

[39] Zhang E, Feng X, Liu F, Zhang P, Liang J, Tang X. Roles of PI3K/Akt and c-Jun signaling pathways in human papillomavirus type 16 oncoprotein-induced HIF-1alpha, VEGF, and IL-8 expression and in vitro angiogenesis in non-small cell lung cancer cells. PLoS One. 2014;9: e103440 10.1371/journal.pone.0103440 25058399 PMC4110025

[40] Cao Y, Linden P, Farnebo J, Cao R, Eriksson A, Kumar V, et al Vascular endothelial growth factor C induces angiogenesis in vivo. Proc Natl Acad Sci U S A. 1998;95: 14389–14394. 10.1073/pnas.95.24.14389 9826710 PMC24383

[41] Tang H, Hao Q, Fitzgerald T, Sasaki T, Landon EJ, Inagami T. Pyk2/CAKβ tyrosine kinase activity-mediated angiogenesis of pulmonary vascular endothelial cells. J Biol Chem. 2002;277: 5441–5447. 10.1074/jbc.M110673200 11739395

[42] Kurashige C, Hosono K, Matsuda H, Tsujikawa K, Okamoto H, Majima M. Roles of receptor activity‐modifying protein 1 in angiogenesis and lymphangiogenesis during skin wound healing in mice. FASEB J. 2014;28: 1237–1247. 10.1096/fj.13-238998 24308973

[43] Jurisic G, Iolyeva M, Proulx ST, Halin C, Detmar M. Thymus cell antigen 1 (Thy1, CD90) is expressed by lymphatic vessels and mediates cell adhesion to lymphatic endothelium. Exp Cell Res. 2010/06/23. 2010;316: 2982–2992. 10.1016/j.yexcr.2010.06.013 20599951 PMC3398154

[44] Jitariu AA, Cimpean AM, Kundnani NR, Raica M. State of the art paper Platelet-derived growth factors induced lymphangiogenesis: evidence, unanswered questions and upcoming challenges. Arch Med Sci. 2015;1: 57–66. 10.5114/aoms.2015.49217 PMC4379379 25861290

[45] Miyazaki H, Yoshimatsu Y, Akatsu Y, Mishima K, Fukayama M, Watabe T, et al Expression of platelet-derived growth factor receptor β is maintained by Prox1 in lymphatic endothelial cells and is required for tumor lymphangiogenesis. Cancer Sci. 2014;105: 1116–1123. 10.1111/cas.12476 24981766 PMC4462385

[46] Nassar ZD, Hill MM, Parton RG, Francois M, Parat MO. Non-caveolar caveolin-1 expression in prostate cancer cells promotes lymphangiogenesis. Oncoscience. 2015;2: 635–645. 10.18632/oncoscience.180 26328273 PMC4549361

[47] Klotz L, Norman S, Vieira JM, Masters M, Rohling M, Dubé KN, et al Cardiac lymphatics are heterogeneous in origin and respond to injury. Nature. 2015;522: 62–67. 10.1038/nature14483 25992544 PMC4458138

[48] Murakami M, Zheng Y, Hirashima M, Suda T, Morita Y, Ooehara J, et al VEGFR1 tyrosine kinase signaling promotes lymphangiogenesis as well as angiogenesis indirectly via macrophage recruitment. Arterioscler Thromb Vasc Biol. 2008;28: 658–664. 10.1161/ATVBAHA.107.150433 18174461

[49] Kendall RT, Feghali-Bostwick CA. Fibroblasts in fibrosis: Novel roles and mediators. Front Pharmacol. 2014;5 5: 1–13. 10.3389/fphar.2014.00001 24904424 PMC4034148

[50] Furtado MB, Nim HT, Boyd SE, Rosenthal NA. View from the heart: cardiac fibroblasts in development, scarring and regeneration. Development. 2016;143: 387–397. 10.1242/dev.120576 26839342

[51] Wong T, McGrath JA, Navsaria H. The role of fibroblasts in tissue engineering and regeneration. Br J Dermatol. 2007;156: 1149–1155. 10.1111/j.1365-2133.2007.07914.x 17535219

[52] Ina K, Kusugami K, Yamaguchi T, Imada A, Hosokawa T, Ohsuga M, et al Mucosal interleukin-8 is involved in neutrophil migration and binding to extracellular matrix in inflammatory bowel disease. Am J Gastroenterol. 1997;92: 1342–1346. 9260803

[53] Zhang K, Zhang Y-Q, Ai W-B, Hu Q-T, Zhang Q-J, Wan L-Y, et al Hes1, an important gene for activation of hepatic stellate cells, is regulated by Notch1 and TGF-β/BMP signaling. World J Gastroenterol. 2015;21: 878–887. 10.3748/wjg.v21.i3.878 25624721 PMC4299340

[54] Von Gise A, Pu WT. Endocardial and epicardial epithelial to mesenchymal transitions in heart development and disease. Circ Res. 2012;110: 1628–1645. 10.1161/CIRCRESAHA.111.259960 22679138 PMC3427736

[55] Teo Z, Sng MK, Chan JSK, Lim MMK, Li Y, Li L, et al Elevation of adenylate energy charge by angiopoietin-like 4 enhances epithelial–mesenchymal transition by inducing 14-3-3γ expression. Oncogene. 2017;36: 6408–6419. 10.1038/onc.2017.244 28745316 PMC5701092

[56] Jamal S, Schneider RJ. UV-induction of keratinocyte endothelin-1 downregulates E-cadherin in melanocytes and melanoma cells. J Clin Invest. 2002;110: 443–452. 10.1172/JCI13729 12189238 PMC150409

[57] Gravdal K, Halvorsen OJ, Haukaas SA, Akslen LA. A switch from E-cadherin to N-cadherin expression indicates epithelial to mesenchymal transition and is of strong and independent importance for the progress of prostate cancer. Clin Cancer Res. 2007;13: 7003–7011. 10.1158/1078-0432.CCR-07-1263 18056176

[58] Nieto MA. The snail superfamily of zinc-finger transcription factors. Nat Rev Mol Cell Biol. 2002;3: 155–166. 10.1038/nrm757 11994736

[59] Conroy KP, Kitto LJ, Henderson NC. αv integrins: key regulators of tissue fibrosis. Cell Tissue Res. 2016;365: 511–519. 10.1007/s00441-016-2407-9 27139180 PMC5010580

[60] Morikawa M, Derynck R, Miyazono K. TGF-β and the TGF-β Family: Context-Dependent Roles in Cell and Tissue Physiology. Cold Spring Harb Perspect Biol. 2016;8: a021873 10.1101/cshperspect.a021873 27141051 PMC4852809

[61] Takeda N, Manabe I, Uchino Y, Eguchi K, Matsumoto S, Nishimura S, et al Cardiac fibroblasts are essential for the adaptive response of the murine heart to pressure overload. J Clin Invest. 2010;120: 254–265. 10.1172/JCI40295 20038803 PMC2798693

[62] Shindo T, Manabe I, Fukushima Y, Tobe K, Aizawa K, Miyamoto S, et al Krüppel-like zinc-finger transcription factor KLF5/BTEB2 is a target for angiotensin II signaling and an essential regulator of cardiovascular remodeling. Nat Med. 2002;8: 856–863. 10.1038/nm738 12101409

[63] Muraoka N, Nara K, Tamura F, Kojima H, Yamakawa H, Sadahiro T, et al Role of cyclooxygenase-2-mediated prostaglandin E2-prostaglandin E receptor 4 signaling in cardiac reprogramming. Nat Commun. 2019;10: 674 10.1038/s41467-019-08626-y 30787297 PMC6382796

[64] Tammela T, Alitalo K. Lymphangiogenesis: Molecular Mechanisms and Future Promise. Cell. 2010;140: 460–476. 10.1016/j.cell.2010.01.045 20178740

[65] Moore-Morris T, Cattaneo P, Puceat M, Evans SM. Origins of cardiac fibroblasts. J Mol Cell Cardiol. 2016;91: 1–5. 10.1016/j.yjmcc.2015.12.031 26748307 PMC4764439

[66] Uosaki H, Fukushima H, Takeuchi A, Matsuoka S, Nakatsuji N, Yamanaka S, et al Efficient and Scalable Purification of Cardiomyocytes from Human Embryonic and Induced Pluripotent Stem Cells by VCAM1 Surface Expression. PLoS One. 2011;6: e23657 10.1371/journal.pone.0023657 21876760 PMC3158088

[67] Iwamiya T, Matsuura K, Masuda S, Shimizu T, Okano T. Cardiac fibroblast-derived VCAM-1 enhances cardiomyocyte proliferation for fabrication of bioengineered cardiac tissue. Regen Ther. 2016;4: 92–102. 10.1016/j.reth.2016.01.005 31245492 PMC6581822

[68] Kwee L, Baldwin HS, Shen HM, Stewart CL, Buck C, Buck C a, et al Defective development of the embryonic and extraembryonic circulatory systems in vascular cell adhesion molecule (VCAM-1) deficient mice. Development. 1995;121: 489–503. 7539357 10.1242/dev.121.2.489

[69] Yang JT, Rayburn H, Hynes RO. Cell adhesion events mediated by alpha 4 integrins are essential in placental and cardiac development. Development. 1995;121: 549–560. 7539359 10.1242/dev.121.2.549

[70] Trincot CE, Xu W, Zhang H, Kulikauskas MR, Caranasos TG, Jensen BC, et al Adrenomedullin Induces Cardiac Lymphangiogenesis after Myocardial Infarction and Regulates Cardiac Edema Via Connexin 43. Circ Res. 2019;124: 101–113. 10.1161/CIRCRESAHA.118.313835 30582443 PMC6318063

[71] Ma F, Li Y, Jia L, Han Y, Cheng J, Li H, et al Macrophage-stimulated cardiac fibroblast production of IL-6 is essential for TGF beta/Smad activation and cardiac fibrosis induced by angiotensin II. PLoS One. 2012;7: e35144 10.1371/journal.pone.0035144 22574112 PMC3344835

[72] Krainock M, Toubat O, Danopoulos S, Beckham A, Warburton D, Kim R. Epicardial Epithelial-to-Mesenchymal Transition in Heart Development and Disease. Brown DL, editor. J Clin Med. 2016;5: 27 10.3390/jcm5020027 26907357 PMC4773783

[73] Widyantoro B, Emoto N, Nakayama K, Anggrahini DW, Adiarto S, Iwasa N, et al Endothelial cell-derived endothelin-1 promotes cardiac fibrosis in diabetic hearts through stimulation of endothelial-to-mesenchymal transition. Circulation. 2010;121: 2407–2418. 10.1161/CIRCULATIONAHA.110.938217 20497976

[74] Wang X, Guo Z, Ding Z, Khaidakov M, Lin J, Xu Z, et al Endothelin-1 upregulation mediates aging-related cardiac fibrosis. J Mol Cell Cardiol. 2015;80: 101–109. 10.1016/j.yjmcc.2015.01.001 25584774

[75] Laine GA, Allen SJ. Left Ventricular Myocardial Edema. Circ Res. 1991;68: 1713–1721. 10.1161/01.res.68.6.1713 2036720

[76] Laine GA, Granger HJ. Microvascular, interstitial, and lymphatic interactions in normal heart. Am J Physiol Circ Physiol. 1985;249: H834–H842. 10.1152/ajpheart.1985.249.4.H834 4051019

[77] Tang T-T, Yuan J, Zhu Z-F, Zhang W-C, Xiao H, Xia N, et al Regulatory T cells ameliorate cardiac remodeling after myocardial infarction. Basic Res Cardiol. 2012;107: 232 10.1007/s00395-011-0232-6 22189560

[78] Brakenhielm E, Alitalo K. Cardiac lymphatics in health and disease. Nat Rev Cardiol. 2019;16: 56–68. 10.1038/s41569-018-0087-8 30333526

[79] El-Chemaly S, Pacheco-Rodriguez G, Ikeda Y, Malide D, Moss J. Lymphatics in idiopathic pulmonary fibrosis: new insights into an old disease. Lymphat Res Biol. 2009;7: 197–203. 10.1089/lrb.2009.0014 20143918 PMC2883488

[80] Hall KL, Volk-Draper LD, Flister MJ, Ran S. New model of macrophage acquisition of the lymphatic endothelial phenotype. PLoS One. 2012;7 10.1371/journal.pone.0031794 22396739 PMC3292559

[81] Kobayashi S, Kishimoto T, Kamata S, Otsuka M, Miyazaki M, Ishikura H. Rapamycin, a specific inhibitor of the mammalian target of rapamycin, suppresses lymphangiogenesis and lymphatic metastasis. Cancer Sci. 2007;98: 726–733. 10.1111/j.1349-7006.2007.00439.x 17425689 PMC11158643

