# Supplementary material for: Human cardiac fibroblasts expressing VCAM1 improve heart function in postinfarct heart failure rat models by stimulating lymphangiogenesis
Source: PLoS One. 2020 Sep 16;15(9):e0237810. doi: 10.1371/journal.pone.0237810 (PMC7494079; doi:10.1371/journal.pone.0237810)
Supplement: S1 Table — (DOCX) [file pone.0237810.s001.docx]

**S Table. 1. Antibodies and reagents used for MACS, FACS, vascular formation assay, and immunohistochemistry**

| Product | Supplier | Reference | Application |
| --- | --- | --- | --- |
| CD106 (VCAM-1)-Biotin | Miltenyi Biotec | 130-104-123 | MACS |
| Anti-Biotin MicroBeads | Miltenyi Biotec | 130-090-485 | MACS |
| CD90-PE | Miltenyi Biotec | 130-114-860 | FACS |
| Vimentin-APC | Miltenyi Biotec | 130-118-361 | FACS |
| α−SMA-APC | R&D Systems | IC1420A | FACS |
| DDR2/TYRO01 | NSJ Bioreagents | F50688-0.4ML | FACS |
| Fibronectin | Abcam | ab2413 | FACS |
| pan Cadherin | Abcam | ab6528 | FACS |
| Cardiac Troponin T | Thermo Fisher Scientific | MA5-12960 | FACS |
| α-Actinin-APC | Assaypro | 33287-05161 | FACS |
| CD29-APC | Miltenyi Biotec | 130-118-122 | FACS |
| CD44-PE | BD Biosciences | 51-9007656 | FACS |
| CD73-APC | BD Biosciences | 51-9007649 | FACS |
| CD105-PerCP-Cy5.5 | BD Biosciences | 51-9007648 | FACS |
| CD106-BV421 | BD Biosciences | 744309 | FACS |
| CD140a-APC | Miltenyi Biotec | 130-115-338 | FACS |
| hMSC Negative Cocktail- PE | BD Biosciences | 51-9007661 | FACS |
| STRO-1 | R&D Systems | FAB 1038G-100UG | FACS |
| CD117-APC | Miltenyi Biotec | 130-111-671 | FACS |
| CD166-APC | Miltenyi Biotec | 130-106-619 | FACS |
| CD338 (ABCG2)-APC | Miltenyi Biotec | 130-105-011 | FACS |
| CD46-APC | Miltenyi Biotec | 130-104-558 | FACS |
| NKX2.5 | Cell Signaling Technology | 8792S | FACS |
| Islet-1 | abcam | ab109517 | FACS |
| GATA4-Alexa Flor 647 | abcam | ab194072 | FACS |
| WT1 | Santa Cruz Biotechnology | sc-7385 | FACS |
| TBX18 | abcam | ab115262 | FACS |
| MEF2C | abcam | ab197070 | FACS |
| CD54 (ICAM-1)-APC | Miltenyi Biotec | 130-103-910 | FACS |
| CD31-APC | Miltenyi Biotec | 130-110-670 | FACS |
| CD144 (VE-Cadherin)-APC | Miltenyi Biotec | 130-100-708 | FACS |
| Mouse IgG1, k Isotype Control- BV421 | BD Biosciences | 562438 | FACS |
| Donkey anti-rabbit IgG- Alexa Fluor 647 | BioLegend, CA | 406414 | FACS |
| Mouse IgG2a, k Isotype Control- Alexa Fluor 647 | BD Biosciences | 557715 | FACS |
| Rabbit IgG, Isotype Control-APC | R&D Systems, MN | IC105A | FACS |
| REA control (S)-PE | Miltenyi Biotec | 130-113-438 | FACS |
| REA Control (S)-APC | Miltenyi Biotec | 130-113-434 | FACS |
| Goat anti-mouse IgG-APC | BioLegend | 405308 | FACS |
| Podoplanin-PE | Santa Cruz Biotechnology | SC-376695 | FACS |
| Mouse IgG2a-PE | Miltenyi Biotec | 130-113-834 | FACS |
| Vimentin | Abcam | ab20346 | Vascular formation assay |
| VE-Cadherin | Abcam | ab33168 | Vascular formation assay |
| Goat Anti-Mouse IgG-Alexa Fluor 647 | Abcam | ab150115 | Vascular formation assay |
| Goat Anti-Rabbit IgG-Alexa Fluor 488 | Abcam | ab150077 | Vascular formation assay |
| Hoechst 33258 solution | Dojindo | 343-07961 | Vascular formation assay |
| Von Willebrand Factor | Abcam | ab6994 | Immunohistochemistry |
| PROX1 | Proteintech | 11067-2-AP | Immunohistochemistry |
| Cardiac Troponin T | abcam | ab8295 | Immunohistochemistry |
| Goat Anti-Rabbit IgG-Alexa Fluor 647 | Abcam | ab150079 | Immunohistochemistry |
| Goat Anti-Mouse IgG-Alexa Fluor 488 | Abcam | ab150113 | Immunohistochemistry |
| ProLong Gold Antifade reagent with DAPI | Thermo Fisher | P36931/ P36935 | Immunohistochemistry |
