# Supplementary material for: Human cardiac fibroblasts expressing VCAM1 improve heart function in postinfarct heart failure rat models by stimulating lymphangiogenesis
Source: PLoS One. 2020 Sep 16;15(9):e0237810. doi: 10.1371/journal.pone.0237810 (PMC7494079; doi:10.1371/journal.pone.0237810)
Supplement: S2 Table — The list is sorted in descending order of the loading scores as listed in the PCA output. (DOCX) [file pone.0237810.s002.docx]

**S Table. 2. List of genes differentially expressed in VCFs compared with VNCFs (limited to the top 1% of loading scores) that are referenced in “cardiovascular system development” and in “heart failure” gene sets.** The list is sorted in descending order of the loading scores as listed in the PCA output.

|  | Loading score |
| --- | --- |
| MYLK | 0.021968191 |
| THY1 | 0.021967963 |
| NR2F2 | 0.021967191 |
| NRP1 | 0.021964903 |
| PDGFRB | 0.021960215 |
| EGR1 | 0.02196008 |
| TBX3 | 0.02195888 |
| CAV1 | 0.021948574 |
| JUN | 0.021948046 |
| VEGFC | 0.021943992 |
| PTK2B | 0.021936365 |
| RAMP1 | 0.021927139 |
| FLT1 | 0.021916286 |
| KLF5 | −0.021895201 |
| CCL2 | −0.021905547 |
| ACTA2 | −0.021916635 |
| SMAD6 | −0.021920648 |
| SMAD7 | −0.02192319 |
| ALDH1A2 | −0.021931304 |
| TGFBR2 | −0.021943793 |
| TEK | −0.021945122 |
| ANGPTL4 | −0.021952854 |
| EDN1 | −0.02195339 |
| PECAM1 | −0.021953734 |
| WT1 | −0.021954026 |
| TMEM204 | −0.021954632 |
| ITGAV | −0.021957482 |
| CXCL8 | −0.021959245 |
| KDR | −0.021959961 |
| PTGS2 | −0.021962064 |
| COL4A1 | −0.021964225 |
| LIF | −0.021967659 |
| TGFBI | −0.021967705 |
| CDH2 | −0.021969496 |
| HES1 | −0.021969566 |
| COL5A1 | −0.021972074 |
| COL4A2 | −0.021980078 |
