# Supplementary material for: Human cardiac fibroblasts expressing VCAM1 improve heart function in postinfarct heart failure rat models by stimulating lymphangiogenesis
Source: PLoS One. 2020 Sep 16;15(9):e0237810. doi: 10.1371/journal.pone.0237810 (PMC7494079; doi:10.1371/journal.pone.0237810)
Supplement: S3 Table — (DOCX) [file pone.0237810.s003.docx]

**S Table. 3. Echocardiography values and corresponding statistical analysis**

|  | **Group** | **0w** | **2w** | **4w** | **6w** | **8w** | **10w** | **12w** | **14w** | **16w** | **18w** |
| --- | --- | --- | --- | --- | --- | --- | --- | --- | --- | --- | --- |
| **LVEF (%)** | Sham | 88.70 ± 0.64** | 92.58 ± 0.63** | 90.82 ± 0.43** | 93.40 ± 1.07** | 90.85 ± 0.52** | 91.12 ± 0.81** | 90.58 ± 0.90** | 90.27 ± 0.76** | 90.22 ± 0.71** | 90.85 ± 0.59** |
|  | Cont. | 52.93 ± 1.26^††^ | 48.85 ± 0.85^††^ | 47.03 ± 0.26^††^ | 44.50 ± 0.45^††^ | 42.20 ± 0.96^††^ | 39.83 ± 1.23^††^ | 38.08 ± 1.45^††^ | 39.55 ± 1.63^††^ | 39.93 ± 0.98^††^ | 38.40 ± 0.41^††^ |
|  | + VCF | 47.70 ± 1.77^††^ | 48.63 ± 2.83^††^ | 54.33 ± 2.52^††^** | 60.53 ± 1.08^††^** | 62.35 ± 2.06^††^** | 63.70 ± 3.57^††^** | 65.38 ± 5.07^††^** | 63.63 ± 6.10^††^** | 66.25 ± 5.43^††^** | 64.35 ± 6.15^††^** |
| **LVFS (%)** | Sham | 51.62 ± 0.89** | 57.90 ± 1.17** | 54.95 ± 0.78** | 60.08 ± 2.23** | 55.00 ± 0.90** | 55.65 ± 1.48** | 54.68 ± 1.43** | 54.18 ± 1.29** | 54.00 ± 1.11** | 55.15 ± 0.95** |
|  | Cont. | 22.23 ± 0.68^††^ | 20.05 ± 0.44^††^ | 19.10 ± 0.11^††^ | 17.80 ± 0.23^††^ | 16.73 ± 0.47^††^ | 15.58 ± 0.58^††^ | 14.78 ± 0.68^††^ | 15.45 ± 0.78^††^ | 15.65 ± 0.44^††^ | 14.90 ± 0.20^††^ |
|  | + VCF | 19.48 ± 0.88^††^ | 20.00 ± 1.48^††^ | 23.08 ± 1.37^††^* | 26.68 ± 0.65^††^** | 27.85 ± 1.31^††^** | 28.90 ± 2.43^††^** | 30.35 ± 3.74^††^** | 29.48 ± 4.57^††^** | 31.10 ± 4.08^††^** | 29.90 ± 4.35^††^** |
| **LVESV (µL)** | Sham | 25.17 ± 1.87** | 18.67 ± 1.93** | 25.67 ± 1.80** | 21.17 ± 4.08** | 30.33 ± 1.50** | 31.50 ± 3.43** | 35.17 ± 4.55** | 38.33 ± 3.32** | 38.67 ± 3.42** | 38.17 ± 2.43** |
|  | Cont. | 197.75 ± 18.27^††^ | 276.50 ± 13.73^††^ | 263.00 ± 22.64^††^ | 337.25 ± 20.74^††^ | 380.00 ± 39.21^††^ | 418.50 ± 56.24^††^ | 463.00 ± 70.24^††^ | 426.50 ± 48.75^††^ | 437.75 ± 81.40^††^ | 469.00 ± 108.60^††^ |
|  | + VCF | 233.75 ± 15.99^††^ | 325.50 ± 33.72^††^ | 298.50 ± 35.03^††^ | 273.50 ± 25.78^††^ | 262.25 ± 31.55^††^* | 239.25 ± 44.93^††^* | 233.25 ± 53.29^††^* | 273.75 ± 67.78^††^* | 242.00 ± 63.86^††^* | 278.75 ± 77.27^††^* |
| **LVEDV (µL)** | Sham | 221.83 ± 5.89** | 249.33 ± 8.23** | 279.00 ± 8.82** | 312.83 ± 12.42** | 333.50 ± 10.72** | 350.83 ± 9.17** | 368.00 ± 14.45** | 393.17 ± 7.39** | 393.17 ± 7.39** | 402.00 ± 3.36** |
|  | Cont. | 423.25 ± 46.56^††^ | 540.25 ± 22.90^††^ | 496.25 ± 41.74^††^ | 607.25 ± 33.38^††^ | 655.25 ± 60.61^††^ | 691.25 ± 81.36^††^ | 743.25 ± 103.15^††^ | 701.50 ± 65.47^††^ | 727.50 ± 50.53^††^ | 761.25 ± 44.04^††^ |
|  | + VCF | 447.50 ± 28.97^††^ | 630.50 ± 44.04^††^* | 650.50 ± 58.98^††^ | 689.75 ± 50.38^††^ | 690.25 ± 51.53^††^ | 642.75 ± 64.67^††^ | 650.50 ± 79.65^††^ | 712.00 ± 88.11^††^ | 683.00 ± 87.09^††^ | 739.25 ± 88.28^††^ |
| **LVAWd**  **(mm)** | Sham | 1.47 ± 0.05 | 1.51 ± 0.02 | 1.45 ± 0.01 | 1.51 ± 0.01** | 1.53 ± 0.01** | 1.53 ± 0.01** | 1.55 ± 0.02* | 1.56 ± 0.02* | 1.57 ± 0.02* | 1.56 ± 0.02* |
|  | Cont. | 1.22 ± 0.06 | 1.04 ± 0.03 | 1.00 ± 0.02 | 1.00 ± 0.02^††^ | 0.98 ± 0.04^††^ | 0.92 ± 0.05^††^ | 0.84 ± 0.02^†^ | 0.86 ± 0.02^†^ | 0.84 ± 0.02^†^ | 0.80 ± 0.03^†^ |
|  | + VCF | 1.42 ± 0.12 | 1.32 ± 0.12 | 1.48 ± 0.27 | 1.28 ± 0.10^†^** | 1.46 ± 0.12** | 1.64 ± 0.20** | 1.84 ± 0.30** | 1.76 ± 0.33** | 1.72 ± 0.37* | 1.76 ± 0.39* |
| **LVPWd**  **(mm)** | Sham | 1.49 ± 0.04 | 1.57 ± 0.03 | 1.48 ± 0.02 | 1.56 ± 0.03 | 1.49 ± 0.02 | 1.64 ± 0.09* | 1.56 ± 0.02* | 1.56 ± 0.05 | 1.56 ± 0.05* | 1.59 ± 0.06 |
|  | Cont. | 1.38 ± 0.02 | 1.44 ± 0.07 | 1.42 ± 0.04 | 1.40 ± 0.02 | 1.50 ± 0.02 | 1.36 ± 0.07^†^ | 1.20 ± 0.06^†^ | 1.38 ± 0.06 | 1.28 ± 0.06^†^ | 1.42 ± 0.04 |
|  | + VCF | 1.60 ± 0.25 | 1.48 ± 0.18 | 1.54 ± 0.14 | 1.82 ± 0.09** | 1.84 ± 0.09** | 1.82 ± 0.09* | 1.84 ± 0.17** | 1.80 ± 0.10** | 1.66 ± 0.08** | 1.66 ± 0.04* |
| **HR**  **(bpm)** | Sham | 356.83 ± 5.91 | 372.17 ± 7.96 | 357.83 ± 8.41 | 363.33 ± 8.10 | 365.50 ± 8.43 | 357.67 ± 12.67 | 354.00 ± 10.11 | 355.00 ± 8.95 | 358.83 ± 11.71 | 355.83 ± 14.94 |
|  | Cont. | 363.25 ± 12.25 | 360.75 ± 11.27 | 355.75 ± 9.49 | 345.75 ± 12.98 | 347.25 ± 7.94 | 351.25 ± 17.51 | 354.25 ± 17.48 | 366.00 ± 15.00 | 359.00 ± 12.50 | 343.50 ± 11.84 |
|  | + VCF | 349.00 ± 6.00 | 334.25 ± 12.46 | 353.5 ± 11.69 | 326.00 ± 11.09 | 337.00 ± 16.25 | 325.75 ± 2.25 | 329.50 ± 4.97 | 327.75 ± 7.25 | 319.25 ± 11.85 | 341.00 ± 8.25 |

Cont, control; VCF, VCAM1-expressing cardiac fibroblast; LVEF, left ventricular ejection fraction; LVFS, left ventricular fractional shortening; LVESV, left ventricular end-systolic volume; LVEDV, left ventricular end-diastolic volume; LVAWd, left ventricular end-diastolic anterior wall thickness; LVPWd, left ventricular end-diastolic posterior wall thickness; HR, heart rate. ††p < 0.01, †p < 0.05 versus sham-treated group, **p < 0.01, *p < 0.05 versus vehicle control group.
