# Supplementary material for: Human cardiac fibroblasts expressing VCAM1 improve heart function in postinfarct heart failure rat models by stimulating lymphangiogenesis
Source: PLoS One. 2020 Sep 16;15(9):e0237810. doi: 10.1371/journal.pone.0237810 (PMC7494079; doi:10.1371/journal.pone.0237810)
Supplement: S1 Fig — Markers of lymphatic endothelial cells are detected in a large majority of HMVEC-Cs. Gray peaks correspond to the expression pattern of isotype control. (DOCX) [file pone.0237810.s004.docx]

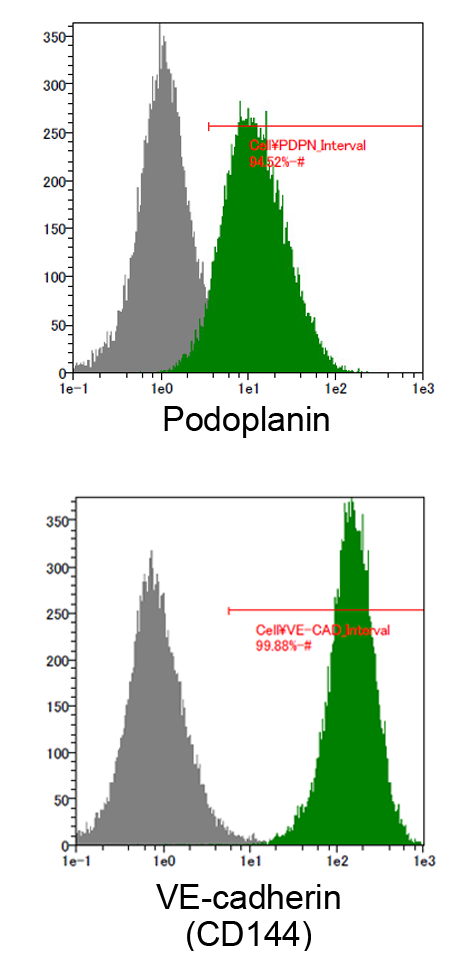


**S Fig. 1. Flowcytometry analysis of HMVEC-Cs.** Markers of lymphatic endothelial cells are detected in a large majority of HMVEC-Cs. Gray peaks correspond to the expression pattern of isotype control.
