# Supplementary material for: Human cardiac fibroblasts expressing VCAM1 improve heart function in postinfarct heart failure rat models by stimulating lymphangiogenesis
Source: PLoS One. 2020 Sep 16;15(9):e0237810. doi: 10.1371/journal.pone.0237810 (PMC7494079; doi:10.1371/journal.pone.0237810)
Supplement: S2 Fig — In each M-mode image, the long light blue dotted line shows the measurement position at the diastolic phase, and the short light blue dotted line shows the measurement position at the systolic phase. (DOCX) [file pone.0237810.s005.docx]

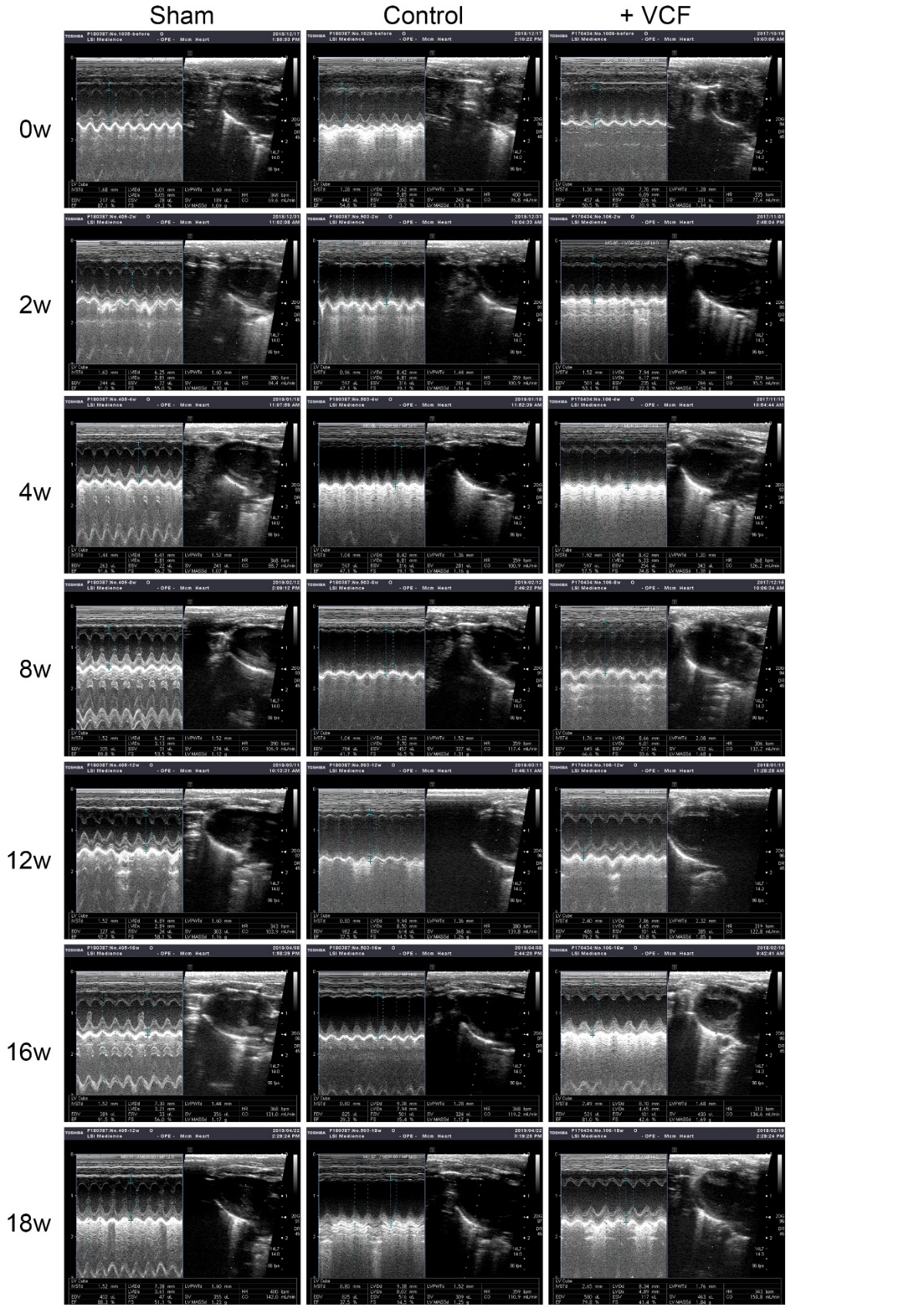


**S Fig. 2. Representative echocardiographic images at each monitoring point (0 weeks: pre-cell administration).** In each M-mode image, the long light blue dotted line shows the measurement position at the diastolic phase, and the short light blue dotted line shows the measurement position at the systolic phase.
