# Supplementary material for: Human cardiac fibroblasts expressing VCAM1 improve heart function in postinfarct heart failure rat models by stimulating lymphangiogenesis
Source: PLoS One. 2020 Sep 16;15(9):e0237810. doi: 10.1371/journal.pone.0237810 (PMC7494079; doi:10.1371/journal.pone.0237810)
Supplement: S1 Text — (DOCX) [file pone.0237810.s006.docx]

## S Text. 1. Whole transcriptome analysis

### RNA extraction, library preparation, and sequencing

Total RNA of cardiac fibroblasts was extracted using a QIAshredder and the RNeasy Plus Mini Kit following the manufacturer’s instructions (Qiagen, Venlo, Netherlands). Sequencing and initial data analysis were performed by GENEWIZ (South Plainfield, NJ). Briefly, RNA concentration and integrity were determined using a NanoDrop One (Thermo Fisher Scientific) and an Agilent Bioanalyzer 2100 (Agilent, Santa Clara, CA). From these extracts, 1 µg of RNA (RNA integrity number (RIN) > 7) was converted into libraries using the NEBNext Ultra RNA Library Prep Kit for Illumina (New England Biolabs, Ipswich, MA). Libraries were sequenced on an Illumina HiSeq 2500 System (Illumina; TruSeq SBS Kit v4-HS, San Diego, CA) in a paired-end 2 × 150 bp configuration.

### Mapping and gene expression evaluation

Technical sequences were removed using Trimmomatic (v0.30). The reference genome was indexed (Ensembl database hg38) and the readings were aligned using HISAT2 (v2.0.1). Alternative splicing was analyzed with ASprofile v1.0.4. Novel transcripts were predicted with Cuffcompare (part of Cufflinks v2.2.1). Single nucleotide variants were identified using samtools v0.1.18 (with command mpileup and Bcftools v0.1.19). Gene and isoform expression was estimated with HTSEQ (v0.6.1). Differential expression was determined with DESeq2 (v1.6.3; Bioconductor package; Padj < 0.05 (BH procedure)). Differential exon usage was determined using DEXSeq (v1.20.1; Bioconductor package).

### Transcriptome analysis

For all transcripts, the ratio of expression in VCFs to that in VNCFs (expressed in FPKM; averages of three samples for each category) was calculated. Results were converted to log_2_FC values. Transcripts presenting |log_2_FC| ≥ 1 and p ≤ 0.05 were classified as differentially expressed. The corresponding heat map was made using Cluster 3.0 and Java TreeView (v. 1.1.6r4). The PCA of differentially expressed genes was realized using the function prcomp in R (v. 4.0.0).
