# Supplementary material for: Human cardiac fibroblasts expressing VCAM1 improve heart function in postinfarct heart failure rat models by stimulating lymphangiogenesis
Source: PLoS One. 2020 Sep 16;15(9):e0237810. doi: 10.1371/journal.pone.0237810 (PMC7494079; doi:10.1371/journal.pone.0237810)
Supplement: S2 Text — (DOCX) [file pone.0237810.s007.docx]

## S Text. 2. Animal experiments

### Animal care and use

Animal experiments were performed by LSI Medience (Tokyo, Japan) in strict accordance with the recommendations of the Guide for the Care and Use of Laboratory Animals of the National Research Council. F344/N Jcl-rnu/rnu rats were purchased from Japan SLC (Shizuoka, Japan). All efforts were made to minimize animal suffering.

### Surgical procedures

Myocardial infarction was induced in male rats (9 weeks old, 133.4-175.0 g) by surgical ligation of the left anterior descending (LAD) artery. Animals were restrained in a supine position and anesthetized by inhalation of 2% isoflurane. Buprenorphine (analgesic; Otsuka Pharmaceutical) was injected subcutaneously at a dose of 0.01 mg/kg, and chest fur was shaved. Under artificial respiration (1%–2% isoflurane, rate: 60 strokes/min, tidal volume: 10–15 ml/kg), thoracotomy was performed by cutting through the costal cartilage of the left third to fifth ribs. After the surgical field was opened with a spreader, the pericardial membrane was stripped to expose the heart. A 6-0 nylon suture was then placed under the LAD artery, and the ends of the string were threaded through a small polyethylene tube (PE50; BD) to form a snare for reversible occlusion. After 30 minutes of ischemia, reperfusion was performed by loosening the snare. After reperfusion, the muscles and skin were sutured. One week after the operation, the cardiac function baseline was acquired by echocardiography, and rats presenting an LVEF inferior to 55% were considered to be representative of heart failure. Selected animals underwent the cell injection protocol with a new thoracotomy (identical procedure). Animals were distributed into three groups following different experimental protocols: injection of human VCFs (+ VCF; 2 × 10^6^ cells in 50 µl of DMEM supplemented with NBCS at 10% v/v; n = 4), injection of vehicle (control group; 50 µl of DMEM supplemented with NBCS at 10% v/v; n = 4), and no injection (sham; n = 6). As the infarcts were large, doses were delivered to two positions (i.e., two injections of 25 µl each) on the border of the scar. No mortality due to cell administration was observed.

After injection, cardiac function was monitored by echocardiography every two weeks for 18 weeks. At the end of the monitoring period, animals were sacrificed and tissue samples were collected.
